# Comparative effectiveness of statins, metformin, and vitamin D, for prevention of preeclampsia: a systematic review and network meta-analysis of randomized trials

**DOI:** 10.1186/s12884-026-09920-6

**Published:** 2026-10-02

**Authors:** Menna Alaa El-Khouly, Mazen Mohammed Mahgoub, Mahmoud E. Abd-Elkareem, Noreen M. El-Bayaa, Jana M. Farouk, Mariam M. Fouad, Habiba Mohammed Salah, Ahmed Khaled Saad, Mohammed Al-azab, Rofida Ayman Mohamed, Kholoud Dahshan, Dalia Atef Abouda

**Affiliations:** 1 Faculty of Medicine, Port Said University, Port Said City, Egypt; 2 Faculty of Medicine, Beni-Suef National University, Beni-Suef, Egypt; 3 Faculty of Medicine, Sana’a University, Sana’a, Yemen; 4 Faculty of Medicine, Alexandria University, Alexandria, Egypt

**Keywords:** Statins, Metformin, Vitamin D, Preeclampsia, Systematic Review, Network Meta-analysis

## Abstract

**Background:**

Preeclampsia is a leading cause of maternal and neonatal morbidity and mortality worldwide, yet effective pharmacological preventive strategies remain uncertain. Statins, metformin, and vitamin D, alone or combined with omega-3, have been investigated for potential protective effects, but comparative evidence is limited.

**Objective:**

To compare the effectiveness of statins, metformin, and vitamin D in preventing preeclampsia and related adverse outcomes among high-risk pregnant women.

**Methods:**

A systematic review and network meta-analysis of randomized controlled trials was conducted. PubMed, Scopus, Web of Science, Cochrane, and Epistemonikos were searched from September 11, 2020, to September 11, 2025. Eight trials involving 4,078 high-risk pregnant women were included. Participants were randomized to statins, metformin, or vitamin D at different doses versus placebo. The primary outcome was the incidence of preeclampsia. Secondary outcomes included gestational hypertension, preterm birth, low birth weight, neonatal intensive care unit admission, gestational diabetes, respiratory distress syndrome, and perinatal outcomes. Risk ratios with 95% confidence intervals were calculated, and interventions were ranked using the surface under the cumulative ranking probabilities.

**Results:**

Eight randomized controlled trials were analyzed. Compared with placebo, higher-dose vitamin D supplementation showed associations with lower estimated risks of preeclampsia, particularly vitamin D 4,000 IU/day (risk ratio 0.21, 95% confidence interval 0.06–0.72) and 60,000 IU monthly (risk ratio 0.36, 95% confidence interval 0.19–0.70). These relative effects corresponded to approximate absolute risk differences of 8% and 6%, respectively, based on observed baseline risks, and reflect associations rather than proven causality. Estimates were derived from limited direct evidence within sparse networks. These dose-specific vitamin D regimens had the highest probabilistic ranking for preeclampsia within the network, but these rankings should be interpreted as exploratory rather than definitive. Higher-dose vitamin D was also associated with trends toward lower event rates of preterm births and low-birth-weight infants, although estimates were imprecise. Evidence for statins and metformin was inconsistent, with wide confidence intervals. SUCRA rankings suggested vitamin D as the highest relative ranking probability overall intervention within the network, followed by statins; however, these rankings remain exploratory given the limited network structure. These associations were derived from limited direct evidence within sparse networks and should be interpreted cautiously. Probabilistic rankings and SUCRA values reflect relative positioning within the network rather than comparative effectiveness.

**Conclusion:**

In high-risk pregnancies, higher-dose vitamin D regimens were associated with lower estimated risks of preeclampsia compared with placebo; however, these findings are based on limited, heterogeneous, and partly indirect evidence from sparse networks. The certainty of evidence is low, and the observed effect sizes should not be interpreted as evidence of clinical effectiveness. These findings are best considered hypothesis-generating and are intended to inform the design of future studies rather than guide clinical practice. Evidence for statins and metformin remains limited and inconsistent. Overall, further large, well-designed randomized trials are required before firm clinical recommendations can be made.

**Supplementary Information:**

The online version contains supplementary material available at https://doi.org/10.1186/s12884-026-09920-6.

## Introduction

Preeclampsia, a serious pregnancy complication characterized by new-onset hypertension and proteinuria after 20 weeks of gestation, continues to be one of the leading contributors to maternal and perinatal morbidity and mortality worldwide, estimated between 2%–8% globally [1]. The pathophysiology of this complex condition is due to an impaired remodelling of the spiral arteries, which leads to placental ischemia, leading to a systemic inflammatory response and endothelial dysfunction usually marked by an anti-angiogenic state with an increased ratio of soluble fms-like tyrosine kinase-1 (sFlt-1): placental growth factor (PlGF) [2, 3].

So, clinical consequences are not limited to delivery but translate into lifelong risks for cardiovascular diseases in mothers and predispose offspring to adverse metabolic and neurodevelopmental profiles [4, 5]. Although low-dose aspirin is currently the only recommended prevention for high-risk women [6, 7], its effectiveness against preeclampsia may be restricted to a reduction in preterm-onset disease. This highlights the ongoing need to identify alternative agents targeting different pathogenic pathways. Several drugs have therefore been considered, including pravastatin, metformin, and vitamin D.

Pravastatin has pleiotropic antiangiogenic effects through up-regulation of VEGF and down-regulation of sFlt-1 and has been shown to reduce sFlt-1 secretion and improve endothelial function in human placental and endothelial cells, suggesting a potential mechanism for benefit via modulation of angiogenic imbalance [8–10]. Emerging evidence indicates that vitamin D plays a role in immunomodulation and placental development, influencing placental angiogenesis and potentially contributing to preeclampsia risk and pathophysiology [11, 12]. Metformin has been shown to reduce sFlt-1 and soluble endoglin secretion and improve endothelial dysfunction and vasodilation, suggesting a potential benefit in preventing or treating preeclampsia through modulation of angiogenic and vascular pathways [9, 13].

Recent systematic reviews revealed that there is insufficient evidence regarding the role of statins in the prevention of pre-eclampsia. Metformin has been shown to reduce the frequency of superimposed pre-eclampsia and associated risk factors. Vitamin D supplementation may lower the risk of pre-eclampsia, although it does not appear to have a clear impact on neonatal outcomes [14–16].

While individual meta-analyses have explored these drugs in isolation, there is a critical lack of evidence directly comparing their relative efficacy. Standard pairwise meta-analysis cannot provide a hierarchy of these treatments. Therefore, we conducted this network meta-analysis to simultaneously compare the effectiveness of statins, metformin, and vitamin D (± omega-3) for the prevention of preeclampsia and related adverse outcomes.

## Objectives

To compare the effectiveness of statins, metformin, and vitamin D ± omega-3 in preventing preeclampsia and related adverse outcomes, including gestational hypertension, preterm birth, low birth weight, neonatal intensive care unit (NICU) admission, gestational diabetes, respiratory distress syndrome (RDS), and perinatal outcomes among high-risk pregnant women.

Given the limited number of randomized trials and sparse network structure, this analysis aimed to generate comparative estimates and hypothesis-generating rankings rather than definitive clinical recommendations.

## Methods

### Protocol registration

The review protocol was registered with PROSPERO International prospective register of systematic reviews on 10 September 2025 (CRD420251156323). This systematic review and network meta-analysis were conducted and reported in accordance with the PRISMA 2020 statement and the PRISMA extension for network meta-analyses (PRISMA-NMA) [17].

### Inclusion criteria

- (a) The study design had to be an RCT, with no restrictions on geographical location.
- (b) The publication date had to be up to September 11, 2025.
- (c) The study population had to consist exclusively of pregnant women with identified high-risk pregnancies, as those with maternal, fetal, placental, or obstetric factors known to increase the risk of preeclampsia or related complications including but not limited to prior history of preeclampsia or chronic hypertension; pre-existing maternal comorbidities (e.g., renal disease, diabetes, autoimmune conditions); maternal obesity or vascular disease; advanced maternal age; multiple pregnancy; abnormal placentation or fetal complications; prior adverse obstetric or fetal outcomes; and vitamin D deficiency [18]. Studies enrolling pregnant women classified as high-risk, as defined by the original trial authors, as there is no universally accepted definition of “high-risk pregnancy.”
- (d) The intervention had to involve metformin, statins, vitamin D, or omega-3 at any daily dose, while the control group received either a placebo or different doses of the intervention proposed (e.g., standard or lower doses of vitamin D). Notably, studies in which aspirin was used in the comparator arm were excluded to minimize confounding from co‑interventions known to modify preeclampsia risk.
- (e) eligible studies were required to report one or more of the following outcomes: incidence of pre-eclampsia or severe pre-eclampsia; early-onset pre-eclampsia (< 34 weeks); gestational hypertension; preterm birth (< 37 or < 34 weeks); small for gestational age (SGA)/intrauterine growth restriction (IUGR); perinatal mortality; NICU admission; maternal serious adverse events (e.g., bleeding, hepatic/renal dysfunction), congenital anomalies, or neonatal adverse events. Outcome definitions were accepted as defined by individual trials and were not reclassified, which may contribute to clinical heterogeneity.
- (f) As no universally accepted definition of high-risk pregnancy exists and outcome definitions varied across trials, these differences may contribute to clinical heterogeneity and should be considered when interpreting the results.

### Exclusion criteria

- (a) Study designs other than randomized controlled trials (e.g., abstracts, editorials, reviews, observational studies, case reports, letters, comments).
- (b) Studies involving women with low-risk pregnancies; animal studies; non-English publications; and studies not reporting relevant outcomes.

### Search strategy and study selection

To identify relevant studies, we attempted to conduct a comprehensive search of all English-language published RCTs. Electronic searches were conducted in PubMed, Scopus, Web of Science, the Cochrane Central Register of Controlled Trials, and Epistemonikos, covering the period from September 11, 2020, to September 11, 2025. The search period was restricted to provide a focused update on the most recent randomized evidence because of the recent propagation of this field in recent years. This ensures that the synthesized data reflects current clinical standards for high-risk pregnancy management and avoids the high clinical heterogeneity often associated with older trials. However, this restriction may have excluded earlier high-quality randomized trials that remain clinically relevant. As a result, the synthesized evidence reflects recent data and should be interpreted as an updated evidence snapshot rather than a comprehensive historical synthesis. This may limit generalizability across different eras of obstetric practice.

The PICO is

Population: pregnant woman with high risk

Intervention: Statins or Metformin, Vitamin D ± Omega 3

Comparator: Placebo or Statins or Metformin, Vitamin D ± Omega 3

Outcome: incidence of pre-eclampsia OR severe preeclampsia; early-onset preeclampsia (< 34 weeks); gestational hypertension; preterm birth (< 37, < 34 weeks); SGA/IUGR; perinatal mortality; NICU admission; maternal serious adverse events (bleeding, hepatic/renal dysfunction); congenital anomalies; neonatal adverse events

The search strategy used was: ((preeclampsia OR "pre-eclampsia" OR preeclampsia* OR preeclamptic OR "gestational hypertension")

AND (pregnant* OR "pregnant women" OR antenatal OR "high risk pregnancy" OR "high-risk pregnant*"))

AND

((statin* OR pravastatin OR simvastatin OR atorvastatin OR rosuvastatin OR fluvastatin OR lovastatin OR "HMG-CoA reductase inhibitor*")

OR (metformin)

OR ("vitamin D" OR cholecalciferol OR calcifediol OR ergocalciferol OR "vitamin D3")

OR ("omega 3" OR "omega-3" OR "fish oil" OR EPA OR DHA OR "n-3 fatty acid*"))

AND

("preterm birth" OR "early-onset preeclampsia" OR "gestational hypertension" OR "small for gestational age" OR SGA OR IUGR

OR "perinatal mortality" OR "NICU admission" OR "maternal adverse event*" OR "congenital anomaly*" OR "neonatal adverse event*")

AND

(random* OR "randomized controlled trial" OR "randomised controlled trial" OR "clinical trial")

All database searches were executed on [September 11, 2025]. The full, reproducible search strategies for each database—including specific boolean operators and filters applied—are provided in Additional File 5 to ensure complete transparency.

The screening of studies involved six review authors independently and blindly evaluating the titles and abstracts of all retrieved studies through Rayyan, an online tool used to streamline and facilitate the screening process [19]. Any record deemed eligible by at least one reviewer had its full text collected for further evaluation against the inclusion criteria by 2 different authors. Any disagreements were resolved through discussion, mutual consensus, or consultation with additional authors.

Although omega-3 supplementation was prespecified as an intervention of interest, no eligible trial was identified. Therefore, omega-3 could not be meaningfully incorporated into the network meta-analysis.

### Data extraction

Data was extracted from the included trial reports using a standardized data extraction form created in Google Sheets. Four reviewers independently extracted data, and discrepancies will be resolved by discussion or another two reviewers. Main baseline characteristics included age, BMI, education, ethnicity, gestational age, and medical history of participants. In addition, we extracted items including study characteristics, ethnicity, inclusion criteria, follow-up duration, drug dosages, intervention and comparator details, outcome measurement, outcomes, side effects, adherence, and maternal and neonatal outcomes.

Key data included the number of participants randomized, as well as the number analysed for each study arm. For dichotomous outcomes, we recorded the number of participants experiencing the event in each trial arm. All forms of data were converted into mean and SD [20]. In case of no response, denied provision, or lack of data, the missing data was identified as “not specified.”

### Quality and risk of bias assessment

For the assessment of risk of bias, the Revised Cochrane Risk-of-Bias Tool for Randomized Trials (RoB 2) was used [21], covering multiple domains: randomization process, deviation from intended interventions, bias in measurement of outcomes, bias due to missing outcome data, bias in the selection of reported results, and other potential biases. Each domain was rated as “low risk of bias,” “some concerns,” or “high risk of bias.”

The overall quality assessment of the RCTs was defined as low risk, some concerns, or high risk according to their overall RoB scores. Studies were considered high quality if they showed a low risk of bias in all domains or had only a single domain with some concerns. They were classified as medium risk if they showed some concerns in several domains but had no high risk of bias. Studies were deemed low quality if they demonstrated a high risk of bias in at least one domain or had multiple domains with some concerns.

Three authors independently conducted the critical quality appraisal of the RCTs. Any disputes were resolved through discussion, mutual consensus, or consultation with an additional author.

### Statistical analysis

#### Network meta-analysis framework

Network meta-analyses were conducted within a frequentist random-effects framework using R (version 4.4.3) under Windows 11, in RStudio (version 2025.05.0), employing the netmeta package (version 3.2.0). Binary outcomes were modeled on the logarithmic risk ratio scale. A random-effects model was chosen a priori due to anticipated clinical and methodological heterogeneity across the included trials, such as differences in patient populations (e.g., definition of high-risk pregnancy), intervention protocols, and settings. A common heterogeneity variance (τ^2^) was assumed across all treatment comparisons within each network. All statistical analyses were conducted within a single, consistent software environment to ensure reproducibility.

#### Outcome data

All outcomes included in the network meta-analysis were binary (e.g., preeclampsia, preterm birth, NICU admission). For each study, outcome data were extracted as the number of events and the total number of participants in each intervention group. Treatment effects were summarized using risk ratios (RRs) with corresponding 95% confidence intervals (CIs).

#### Handling of multi-arm trials

Handling of Multi-Arm Trials: Studies with more than two intervention arms (multi-arm trials) were included and appropriately accounted for within the netmeta package framework. This approach preserves the within-study correlation between treatment comparisons originating from the same trial and prevents double-counting of participants in the shared control group.

#### Consistency and inconsistency assessment

Assumption of Consistency and Inconsistency Assessment: The network meta-analysis relies on the assumption of consistency between direct and indirect evidence. Global inconsistency was evaluated using the design-by-treatment interaction model, while local inconsistency was assessed using node-splitting methods where applicable. Given the sparse network structure, tests for inconsistency were underpowered; given the small number of studies and limited closed loops within the networks, statistical tests for inconsistency had low power to detect potential incoherence. Therefore, non-significant results should not be interpreted as evidence of true consistency but rather as reflecting limited ability to detect inconsistency.(design-by-treatment interaction *p* > 0.05).

#### Handling of missing data

Handling of Missing Outcome Data: For the primary analysis, we utilized an available-case approach, using all participants with reported outcome data in each study as provided in the original publications. We did not impute missing outcome data. The potential impact of missing data was considered qualitatively as part of the risk-of-bias assessment.

#### Treatment ranking

Treatments were ranked according to the surface under the cumulative ranking curve (SUCRA). Given the sparse nature of the network, SUCRA values were interpreted as descriptive and hypothesis-generating rather than definitive indicators of treatment superiority. The network was sparse and largely dominated by placebo-controlled trials, with very limited head-to-head comparisons. Treatment rankings and SUCRA values are valid only within each specific connected network and outcome. Rankings derived from different, disconnected networks are not directly comparable and should not be interpreted as indicating relative effectiveness across networks.

#### Network structure and risk of bias

All eligible randomized trials were included in the primary analyses regardless of risk-of-bias assessment to preserve network connectivity. Formal sensitivity analyses excluding studies at high risk of bias were not performed due to the limited number of available trials and the risk of network fragmentation. Nevertheless, the potential influence of study quality on effect estimates was carefully considered during interpretation, and findings based on high-risk studies were interpreted with additional caution.

#### Network geometry

Interventions were defined as separate nodes based on differences in pharmacological class, dosage regimen, and clinical administration protocols. Different vitamin D dosing strategies were treated as separate nodes due to potential differences in biological effect and clinical response across dosage levels, as the relationship between vitamin D dose and clinical outcomes in high-risk pregnancies is not assumed to be linear. Similarly, placebo/no treatment was defined as a single comparator node, while pravastatin 20 mg and metformin 2500 mg were each defined as distinct nodes reflecting their different pharmacological classes and mechanisms of action. This approach was chosen to preserve clinical interpretability and avoid inappropriate pooling of interventions that differ meaningfully in their biological rationale.

However, this node definition had important structural consequences for the network. Most critically, it resulted in two completely disconnected sub-networks: a vitamin D dose-comparison network anchored at vitamin D 400 IU, and a placebo-anchored network including vitamin D 60,000 IU, pravastatin 20 mg, and metformin 2500 mg. Because these two sub-networks share no common comparator node, no direct or indirect evidence bridges them, and cross-network comparisons are therefore not estimable. Additionally, within each sub-network, the granular node definition contributed to network sparsity in some comparisons, particularly where certain dose-to-dose head-to-head trials were unavailable.

#### Contribution of direct and indirect evidence

The contribution of direct and indirect evidence varied across outcomes and networks. Overall, the evidence base was structured around two disconnected sub-networks that could not be bridged, meaning no comparison between them was estimable. The network plots (Fig. 3 in Additional file 2) and forest plots (Fig. 7 in Additional file 2) are provided to allow readers to visually verify the network geometry and the relative contribution of evidence for each outcome and comparison.

Within the vitamin D dose-comparison network (pre-eclampsia, gestational diabetes, preterm birth, and low birth weight), most pairwise comparisons were informed by direct head-to-head evidence. The only exceptions were: vitamin D 1500 IU vs 2000 IU (indirect only, across pre-eclampsia, gestational diabetes, and preterm birth), and vitamin D 800 IU vs 2000 IU and 800 IU vs 4000 IU (indirect only, for low birth weight).

Within the placebo-anchored network, all active interventions present in each outcome network were directly compared against placebo, providing direct evidence for those specific comparisons. However, no head-to-head trials existed between any active interventions, so all comparisons among active treatments — such as vitamin D 60,000 IU vs pravastatin, vitamin D 60,000 IU vs metformin, and pravastatin vs metformin — were informed exclusively by indirect evidence. Notably, vitamin D 60,000 IU was absent from gestational hypertension, NICU admission, and respiratory distress syndrome networks, and pravastatin was absent from the low birth weight network.

Overall, mixed estimates combining direct and indirect evidence arose where multiple paths existed within a network, while purely indirect estimates arose only where no head-to-head trial was available between two specific nodes.

#### Uncertainty and confounding

Effect estimates for metformin and pravastatin were associated with wide confidence intervals, reflecting uncertainty due to small sample sizes and low event rates. Potential confounding by indication was considered in the interpretation of findings.

As this was a study-level network meta-analysis, adjustment for individual-level confounders was not possible. Sensitivity analyses stratified by gestational age at intervention initiation or excluding trials with late initiation could not be performed due to inconsistent reporting of initiation timing and overlapping gestational windows across included trials, which would have led to substantial loss of network connectivity. Potential confounding by baseline risk factors (e.g., obesity, diabetes, and vitamin D level) was therefore addressed qualitatively during interpretation rather than statistically controlled.

### Assessment of publication bias

Formal assessment of publication bias using funnel plots or statistical tests was not performed, as the number of included trials per comparison was small and insufficient to provide reliable or interpretable results. This approach is consistent with methodological guidance for network meta-analyses involving sparse networks.

This decision was made to maximize available evidence and preserve network connectivity in the context of a limited evidence base.

### Certainty of evidence

The certainty of evidence across the network was qualitatively considered using the principles of the Confidence in Network Meta-Analysis (CINeMA) framework, which evaluates within-study bias, across-studies bias, indirectness, imprecision, heterogeneity, and incoherence. A formal CINeMA assessment was not conducted due to the sparse network structure, limited number of trials per comparison, and wide confidence intervals for several outcomes. Overall, the certainty of evidence was judged to be low to very low for most outcomes, primarily due to imprecision, indirectness, and sparse data.

### Assumptions of transitivity and clinical comparability

The validity of network meta-analysis relies on the assumption of transitivity, which requires that included trials be sufficiently comparable with respect to clinical and methodological characteristics that may modify treatment effects.

All included randomized trials enrolled pregnant women classified as high-risk for preeclampsia; however, the criteria for high-risk pregnancy were defined by the original trial investigators and varied across studies. Populations included women with prior preeclampsia, obesity, metabolic comorbidities, or vitamin D deficiency.

To reduce heterogeneity related to co‑interventions, trials in which aspirin was included in the comparator arm were excluded during study selection; however, residual variability in background care across studies cannot be fully excluded.

Importantly, these population differences may not only contribute to between-study heterogeneity but could specifically bias indirect treatment comparisons. For example, the relative benefit of vitamin D supplementation may be amplified in trials enriched for vitamin D-deficient women, while the apparent efficacy of metformin may be inflated in populations with metabolic comorbidities such as obesity or insulin resistance — meaning that indirect comparisons between these interventions via a shared placebo may reflect differences in patient susceptibility rather than true differences in pharmacological efficacy.

Potential effect modifiers, including baseline vitamin D status, gestational age at intervention initiation, dosing regimens, and treatment duration, were inconsistently reported, which may limit the comparability of indirect treatment effects. Despite these limitations, all interventions were compared against a common reference (placebo or low-dose supplementation). Residual violations of transitivity at the individual-patient level cannot be excluded. Results from indirect comparisons should therefore be interpreted with caution, particularly for treatments supported by sparse evidence. Although no clear structural imbalances were identifiable at the study level, the limited reporting of potential effect modifiers and the sparse nature of several comparisons restrict the certainty with which the transitivity assumption can be considered satisfied.

## Results

### Literature search results & study selection

A total of 262 articles were identified through searches of five databases (Scopus = 125, Epistemonikos = 71, Web of Science = 35, PubMed = 21, and Cochrane = 10). After removing 98 duplicates, 164 records remained for title and abstract screening, of which 147 were excluded. Seventeen reports were sought for full-text assessment, all of which were retrievable, and 13 were assessed for eligibility. Five full-text articles were excluded for reasons including ineligible intervention, population not meeting high-risk criteria, or comparator not being PICO-compliant. Ultimately, eight randomized controlled trials were included in the review and subsequent network meta-analysis. All included studies were published in English in various peer-reviewed journals. The literature screening process is summarized in the PRISMA flow diagram (Fig. 1 in Additional file 2).

### Characteristics of included studies

The included studies reported a total of 4078 participants. A total of 2281 participants received the interventions, and 1741 participants received the comparator. The main characteristics of the included studies are summarized in Table 1 in Additional file 1.

### Baseline characteristics of included studies

Eight studies were included in this review (Table 2 in Additional file 1). The mean age of participants varied widely across studies (21.2–34.3 years), although values were generally comparable between intervention and control groups within individual trials. Mean body mass index (BMI) also varied widely across studies (19.7–30.9 kg/m^2^). Gestational age at baseline or at initiation of the intervention varied substantially (approximately 11.7–35.8 weeks). Notably, gestational age was not uniformly defined across studies, as some reported gestational age at intervention initiation, whereas others reported it at the time of assessment, contributing to clinical and methodological heterogeneity.

Baseline serum 25-hydroxyvitamin D [25(OH)D] concentrations varied widely across studies (7.2–39.9 ng/mL), indicating considerable differences in baseline vitamin D status among study populations. Overall, while baseline characteristics were generally comparable within individual studies, the observed variability across trials reflects substantial clinical heterogeneity, which should be considered when interpreting pooled estimates and may partially explain between-study variability in effect sizes.

### Quality assessment of included studies

The Cochrane RoB 2 tool was used to assess the quality of evidence and risk of bias of the included RCTs. As shown in Fig. 2 in Additional file 2, three studies were judged to have a high overall risk of bias, three studies had some concerns, while two studies were rated as low risk of bias.

The overall risk of bias assessment revealed that several trials, particularly those investigating vitamin D supplementation, were rated as having "some concerns" or "high risk of bias," primarily in domains related to deviations from intended interventions and missing outcome data.

### Network meta-analysis modelling approach

A frequentist random-effects network meta-analysis was conducted using the netmeta package in R (version 5.3), assuming a common heterogeneity variance (τ^2^) across all treatment comparisons. Network-level analyses and treatment rankings were generated in R. Network estimates were derived from both direct and indirect evidence.

The assumption of transitivity was assessed qualitatively by examining the distribution of key potential effect modifiers across treatment comparisons, including baseline risk definition for preeclampsia, gestational age at randomization, baseline vitamin D status, and the presence of metabolic comorbidities such as obesity or diabetes. Although these characteristics varied across studies, no clear systematic imbalance was identified that would completely invalidate indirect comparisons.

Nevertheless, this clinical heterogeneity may have influenced the precision of network estimates and should be considered when interpreting treatment rankings. The design-by-treatment interaction model was used to evaluate global inconsistency, and the node-splitting technique was used where appropriate to analyze local inconsistency. τ^2^ and I^2^ statistics were used to quantify between-study heterogeneity. Multi-arm trials were appropriately accounted for within the network meta-analysis framework to avoid double-counting of participants and to preserve within-study correlations.

### Network geometry, evidence sparsity, and robustness of comparisons

Several comparisons in the network are informed by a very limited number of trials, and in some cases, a single study contributes to a given comparison, for which its estimate should be interpreted cautiously. Additionally, some treatment nodes are connected only indirectly. Consequently, treatment effect estimates and ranking probabilities should be interpreted with caution, as sparse evidence may reduce the certainty and robustness of the conclusions. This limitation should be kept in mind when interpreting network estimates across all outcomes.

### Primary maternal outcome

#### Preeclampsia

The network meta-analysis for preeclampsia included four interventions: placebo/no treatment, metformin 2500 mg, pravastatin 20 mg, and specific vitamin D dosing regimens. Separate networks were constructed based on the available comparators (placebo or vitamin D 400 IU) to preserve network connectivity and avoid inappropriate indirect comparisons. The network structure and comparative effect estimates for preeclampsia are illustrated in Figs. 3 and 4 in Additional file 2.

##### Vitamin D dose-comparison network

In comparisons using vitamin D 400 IU as the reference, vitamin D 4,000 IU/day was estimated to be associated with a lower estimated risk of preeclampsia (RR = 0.21, 95% CI: 0.06–0.72). The network comparing different vitamin D doses for preeclampsia included direct comparisons from 4 trials [41–44]. All four included trials were at risk of bias (three high risk and one with some concerns), which may have influenced the estimated treatment effects. Specifically, vitamin D 400 IU vs. 1500 IU and 400 IU vs. 4000 IU were each directly compared in 1 trial; vitamin D 2000 IU vs. 4000 IU was directly compared in 1 trial; and vitamin D 400 IU vs. 2000 IU was not directly compared in any trial; this estimate relies entirely on indirect evidence, as illustrated in the corresponding network plot (Fig. 3A in Additional file 2), due to limited network connectivity and should be cautiously interpreted.

##### Placebo-comparison network

The placebo-referenced network for preeclampsia was informed by 4 trials. Among these, 1 trial [45], which was assessed as having some concerns of bias, directly compared vitamin D 60,000 IU/month with placebo and yielded an estimate consistent with a lower estimated risk (RR = 0.36, 95% CI: 0.19–0.70). The comparisons of pravastatin and metformin versus placebo were each informed by 2 trials and 1 trial, respectively, resulting in wide confidence intervals (pravastatin: RR = 1.03, 95% CI: 0.77–1.37; metformin: RR = 1.98, 95% CI: 0.69–5.73). These estimates should be interpreted in light of limited direct evidence and sparse network connectivity.

##### Heterogeneity assessment

For the primary preeclampsia networks, between-study heterogeneity was minimal, with I^2^ = 0% across both the vitamin D dose-comparison network and the placebo-referenced network. The estimated τ^2^ was equal to zero or not estimable due to sparse data. However, this apparent absence of statistical heterogeneity likely reflects limited statistical power, small numbers of trials, and sparse event counts rather than true clinical homogeneity across interventions.

#### Incidence of preeclampsia in individual studies

Table 3 (Additional file 1) summarizes the incidence of preeclampsia across intervention and control groups in the included studies. Most interventions involved varying doses of vitamin D, with additional trials evaluating pravastatin and metformin.

Across studies comparing vitamin D supplementation with control groups, lower doses (e.g., 400–800 IU) were associated with modest reductions in preeclampsia incidence, whereas higher doses (e.g., 2,000–4,000 IU) showed numerical association with larger estimated reductions, with rates ranging from 1.2% to 2.1% in some intervention arms compared with 5.7% to 24% in control groups. For example, Kabuyanga [45] reported a preeclampsia rate of 2.1% in the 4,000 IU group versus 5.7% in the control group, and Manasova [44] observed a rate of 6.9% in the 4,000/2,000 IU group compared with 24% in the 500 IU control group.

For pharmacological interventions, pravastatin did not show consistent reductions in preeclampsia incidence. Dóbert [46] reported similar rates between pravastatin (14.6%) and placebo (13.6%), while Costantine [47] reported a lower incidence with pravastatin (20% vs. 50% with placebo). Metformin [48] was associated with a preeclampsia rate of 3.8% compared with 1.9% in the placebo group.

### Secondary maternal outcomes

#### Gestational hypertension

The network plot for gestational hypertension comprised three interventions: placebo/no treatment, pravastatin 20 mg, and metformin 2500 mg. All pairwise comparisons in this network were informed by direct evidence, with each comparison informed by a single trial (Costantine 2021 [47] for pravastatin and Dunne 2023 [48] for metformin), which limits the certainty of the estimated effects. Therefore, the estimate should be interpreted cautiously. Neither metformin (RR = 1.14, 95% CI: 0.65–2.01) nor pravastatin (RR = 0.92, 95% CI: 0.45–1.89) demonstrated statistically significant differences compared with placebo. Network plots, comparative effect estimates, and treatment ranking probabilities for gestational hypertension are shown in Figs. 3, 4, and 5 in Additional file 2.

#### Gestational diabetes

Using vitamin D 400 IU as the reference comparator, no vitamin D dosage demonstrated a statistically significant effect on the risk of gestational diabetes, as all confidence intervals included the null value. As the network relied on one trial [41] with a high risk of bias for the available direct comparisons (400 IU vs. 1500 IU and 400 IU vs. 4000 IU), the robustness of the estimated treatment effects may be limited. The effect estimates for other doses are largely derived from indirect evidence. Comparative network estimates and SUCRA-based ranking probabilities for gestational diabetes are presented in Figs. 4 and 5 in Additional file 2. Although SUCRA values suggested variability in ranking probabilities across vitamin D doses, these differences were not supported by statistically significant effect estimates and should therefore be interpreted cautiously.

### Neonatal outcomes

#### Preterm birth

In placebo-referenced comparisons, vitamin D 60,000 IU/month was associated with a lower estimated risk of preterm birth (RR = 0.51, 95% CI: 0.33–0.79). This finding is supported by direct evidence from 1 trial [45]. Metformin 2500 mg showed an imprecise estimate suggesting a possible increase in risk (RR = 1.40, 95% CI: 0.77–2.55), with wide confidence intervals crossing the null. No statistically significant differences in vitamin D dosage regimens were found in dose–response networks using 400 IU of vitamin D as the reference, which were informed by 3 trials [41–43]. Although ranking probabilities differed among dosages, the limited available data should be interpreted cautiously. Xiaomang 2021 [41] and Nausheen 2021 [43] were assessed as having a high risk of bias, while Ku 2024 [42] and Kabuyanga 2024 [45] raised some concerns, which may have influenced the estimated network effects and treatment rankings. Network effect estimates and treatment rankings for preterm birth are illustrated in Figs. 4 and 6 in Additional file 2.

#### NICU admission

No statistically significant differences were observed between interventions for NICU admission. The network was informed by 2 trials [46, 47], which were assessed as having a low risk of bias, both directly comparing pravastatin 20 mg with placebo. Comparative network estimates and ranking probabilities for NICU admission are presented in Figs. 4 and 6 in Additional file 2. Although there was a non-significant trend with pravastatin 20 mg (RR = 0.64, 95% CI: 0.35–1.16), the confidence intervals crossed the null. Although these results are still preliminary, ranking probabilities indicated variation among strategies, without implying clinical effectiveness.

#### Respiratory Distress Syndrome (RDS)

None of the assessed therapies showed statistically significant effects for respiratory distress syndrome. Pravastatin 20 mg showed a numerically lower but non-significant estimate (RR = 0.55, 95% CI: 0.27–1.12). This comparison was informed by two trials [46, 47], which were assessed as having a low risk of bias, directly comparing pravastatin with placebo. Given the limited number of studies, these findings should be interpreted cautiously. Network estimates and SUCRA-based ranking probabilities for respiratory distress syndrome are shown in Figs. 4 and 6 in Additional file 2.

#### Low Birth Weight (LBW)

Compared with placebo/no treatment, vitamin D 60,000 IU/month was associated with a lower estimated risk of low birth weight (RR = 0.43, 95% CI: 0.28–0.68). This estimate is supported by direct evidence from 1 trial [45], which was assessed as having some concerns of bias, potentially influencing the estimated network effects. Effect estimates and treatment ranking probabilities for low birth weight are summarized in Figs. 4 and 6 in Additional file 2. Metformin 2500 mg showed an imprecise estimate, suggesting a possible increase in risk (RR = 1.76, 95% CI: 0.79–3.92), informed by a single trial [48], which was assessed as having a low risk of bias. Therefore, the estimate should be interpreted cautiously.

Within vitamin D dose–response networks, no statistically significant differences were observed among daily dosing regimens. This network was informed by 3 trials [41–43]. Two trials were assessed as having a high risk of bias, and one with some concerns, which may influence the estimated network effects. Ranking probabilities varied across doses but should be considered exploratory.

### Heterogeneity and inconsistency

Network heterogeneity was minimal across all analyses (I^2^ = 0%), and τ^2^ was either equal to zero or not estimable. However, this apparent absence of statistical heterogeneity should not be interpreted as evidence of true clinical homogeneity. Rather, it most likely reflects the small number of included trials, sparse event counts, and limited statistical power to detect between-study variability.

Global inconsistency assessment using the design-by-treatment interaction model did not identify statistically significant disagreement between direct and indirect evidence. Nevertheless, the interpretation of inconsistency results is limited by the sparse network structure and the small number of studies contributing to several comparisons. Consequently, heterogeneity and inconsistency estimates in this network are imprecise, and true underlying clinical heterogeneity may still be present but undetectable. These findings highlight the need for cautious interpretation of treatment rankings and network estimates until larger and more robust datasets become available.

### Treatment ranking/SUCRA

Although SUCRA values provide a probabilistic ranking of interventions, these rankings should not be interpreted as definitive evidence of comparative superiority. In sparse networks, ranking probabilities may appear more certain than the underlying effect estimates and may be influenced by limited data or wide confidence intervals. Therefore, SUCRA rankings in this analysis should be interpreted alongside effect sizes, confidence intervals, and the structure of the evidence network.

### Summary of main findings

Vitamin D 60,000 IU frequently ranked among the relatively higher positions across several outcomes; however, these rankings were derived from a limited number of trials and sparse networks and should be interpreted as descriptive signals rather than evidence of clinical superiority.

Overall, findings suggesting relatively more favorable estimates for higher-dose vitamin D should be interpreted in the context of the underlying risk-of-bias profile, as most contributing trials were assessed as having some concerns or a high risk of bias.

Pravastatin 20 mg appears to show a possible numerical advantage for neonatal NICU admission and RDS, but it was not statistically significant.

In contrast, metformin 2500 mg showed comparatively lower rankings across maternal and neonatal outcomes, which indicate inconclusive evidence and highlight the need for further research. Treatment ranking probabilities derived from SUCRA and visualized using rankograms are presented to support the interpretation of effect estimates and should be regarded as exploratory, particularly in sparse networks and underpowered comparisons.

## Discussion

This systematic review and network meta-analysis synthesized randomized evidence comparing vitamin D, statins, and metformin for the prevention of preeclampsia and related outcomes in high-risk pregnant women. Included trials enrolled heterogeneous high-risk populations, including women with prior preeclampsia, obesity, metabolic comorbidities, or vitamin D deficiency, since they have a greater chance of developing complications during pregnancy and for the newborn [18, 22]. The criteria used to define “high-risk pregnancy” were determined by the individual trial investigators rather than being standardized across studies, resulting in heterogeneous populations that may compromise the transitivity assumption of the network meta-analysis. A key assumption of network meta-analysis is transitivity, which requires that the included studies are sufficiently comparable in terms of clinical and methodological characteristics. In the present analysis, there was notable variability across studies in baseline maternal risk profile, prevalence of obesity and metabolic disorders, probable baseline vitamin D status, gestational age at treatment initiation, dosage regimens, and background antenatal care. These differences may act as effect modifiers and could potentially compromise the validity of indirect comparisons. Therefore, the assumption of transitivity may not have been fully met, and this should be considered when interpreting the network estimates.

Additionally, variations in baseline risk, gestational age at treatment initiation, and duration of interventions across included trials may not only influence treatment effects but also affect the internal validity of the network meta-analysis and limit the generalizability of the findings. For instance, trials enrolling women with a history of preeclampsia tended to report larger reductions in recurrence risk compared to those including women with chronic hypertension or metabolic risk factors, indicating that population definitions may influence treatment effect estimates. Baseline vitamin D status also appears to modify outcomes, with greater reductions in preeclampsia risk observed in participants who were vitamin D deficient at enrollment. Studies initiating supplementation earlier in gestation generally reported larger treatment effects, highlighting gestational age at intervention as a potential source of heterogeneity. Differences in dosing regimens, such as high-dose monthly versus daily supplementation, may account for variability in treatment response across trials.To contextualize the magnitude of effect, it is important to note that relative risk reductions observed in sparse networks may correspond to modest absolute risk differences, particularly when baseline event rates are low. Therefore, clinical interpretation should consider both relative and absolute measures of effect.

Although dosing regimens were analyzed as distinct nodes, pharmacological comparability remains uncertain. Only five of eight trials reported serum 25(OH)D concentrations, and reported values varied markedly across studies, indicating that nominal dose equivalence does not necessarily reflect similar biological exposure.

Because most treatment comparisons in this network were informed primarily by placebo-controlled trials rather than direct head-to-head studies, many relative effect estimates rely substantially on indirect evidence. It is also important to distinguish between estimates derived from direct evidence, indirect evidence, and mixed comparisons. In several comparisons within this network, effect estimates were driven primarily by indirect evidence due to the limited number of head-to-head trials. Indirect comparisons are inherently more uncertain and rely heavily on the transitivity assumption; therefore, these estimates should be interpreted as exploratory.

Such comparisons are inherently more assumption-dependent and may be more vulnerable to bias arising from between-study differences in populations, intervention protocols, and outcome assessment. Therefore, indirect comparisons should be interpreted cautiously and considered hypothesis-generating rather than confirmatory evidence of comparative effectiveness.

A key assumption underlying network meta-analysis is transitivity, which requires that included studies are sufficiently comparable in clinical and methodological characteristics that could modify treatment effects. Although we explored potential effect modifiers such as baseline risk profile, gestational age at treatment initiation, dosing regimens, and baseline vitamin D status, variability across trials suggests that this assumption may only be partially satisfied. Differences in population definitions, intervention timing, and supplementation strategies may have influenced relative treatment effects. These factors may also limit the certainty of indirect comparisons. Consequently, while the network estimates provide useful comparative insights, they should be interpreted as conditional on the plausibility of the transitivity assumption rather than as definitive comparative effectiveness evidence.

Baseline vitamin D deficiency was not included as a covariate or effect modifier in analyses because reporting was inconsistent across trials. Therefore, potential modification of treatment effects by baseline vitamin D status could not be formally assessed.

Notably, no eligible trials directly evaluated combined vitamin D and omega-3 supplementation, limiting conclusions regarding synergistic effects. Overall study quality was moderate, with three trials judged to be at high risk of bias. Although statistical heterogeneity appeared minimal, this likely reflects sparse networks and limited power rather than true clinical homogeneity.

None of the included trials investigated the co-administration of vitamin D and omega-3 fatty acids. Consequently, potential biological interactions between these agents, including synergistic effects on endothelial function or inflammatory pathways, could not be evaluated and remain speculative.

Across trials evaluating vitamin D, associations with improved maternal and fetal outcomes were reported compared with control groups, with higher doses tending to show more favorable estimates. Exploratory treatment rankings (SUCRA) indicated that vitamin D 4,000 IU/day had the highest probability of a favorable ranking within this network; however, these rankings should be interpreted cautiously given the limited and heterogeneous evidence base. Pravastatin demonstrated possible signals of neonatal benefit but remains limited by small and inconsistent data, while metformin shows inconclusive effects.

It is important to emphasize that SUCRA values represent probabilistic rankings within a given evidence network rather than measures of absolute clinical benefit. In small or sparsely connected networks, particularly those with heterogeneous interventions and limited direct evidence, ranking metrics may be unstable and sensitive to small changes in the data particularly where confidence intervals are wide and precision is low. Accordingly, SUCRA rankings in this analysis should not be interpreted as definitive evidence of clinical superiority but rather as exploratory indicators requiring confirmation in adequately powered head-to-head trials.

Vitamin D supplementation, particularly at higher doses, was associated with lower risks of preeclampsia across included trials, specifically 4,000 IU per day, which showed the most favorable association within this network model according to the network analysis. The findings related to vitamin D should be interpreted with particular caution. While some vitamin D regimens appeared favorable in the network estimates and ranking analysis, the underlying evidence was limited, with few direct head-to-head comparisons and a sparse network structure. In addition, treatment effects may depend on baseline vitamin D deficiency status, which was not consistently reported across trials. Therefore, these findings should be viewed as hypothesis-generating rather than definitive evidence of superiority, and they should not be interpreted as sufficient to support changes in clinical practice at this stage.

This is consistent with Rostami et al. [23], who showed a modest but significant prevention of preeclampsia by vitamin D [OR, 0.50 (95% CI, 0.34–0.88)]. The Institute of Medicine previously established 4,000 IU/day as the safe upper intake level during pregnancy [24], and the Society of Endocrinology recommends that 4,100 IU daily (35,000 IU/week) dosing is safe for both mother and infant [25]. There is no evidence for safety in greater amounts, and European and North American guidelines are still conservative at 600 IU/day [24, 26]. The SUCRA ranking was consistent with these findings; however, a SUCRA value of 0.89 for vitamin D 4,000 IU reflects a higher probability of a favorable ranking within this specific network, but does not confirm clinical superiority. This probability should be interpreted cautiously, particularly given the wide confidence intervals, heterogeneous populations, and reliance on indirect comparisons, and does not confirm superiority over other dosages in preventing preeclampsia. In addition, monthly high-dose vitamin D (60,000 IU) had fewer cases of preeclampsia (RR 0.36, 95% CI 0.19–0.70), but there was no clear evidence to be superior to daily low doses (RR 0.21, 95% CI 0.06–0.72). Daily dosing may result in more stable serum levels and potentially more consistent physiological effects; however, this hypothesis was not directly tested within the included trials. In relation to preterm delivery, the previous studies were discordant, but, in our research results, most doses of vitamin D had fewer preterm births, with the 2,000 IU/day dose showing the most favorable ranking probability within this outcome network.

The findings suggest that vitamin D supplementation during early and mid‑pregnancy may be associated with a lower risk of preeclampsia and preterm delivery. Vitamin D deficiency (20–30 ng/mL) and insufficiency (< 20 ng/mL) were also found to be linked with preeclampsia risk [27]. Several meta-analyses confirmed this association [28–32]. A 2015 analysis found no effect of vitamin D supplementation on preeclampsia [33]. This can be attributed to the small sample size of only three trials (*n* = 654), and methodological and clinical differences. Their review predominantly included lower and heterogeneous vitamin D doses and relied on pairwise comparisons only, whereas our network meta-analysis integrates both direct and indirect evidence across higher-dose regimens and more recent RCTs. Differences in baseline vitamin D status, timing of intervention initiation, and regional nutritional profiles may also modify treatment response and contribute to divergent findings. In a second randomized trial of 4,400 vs. 400 IU/day from 10 to 18 weeks’ gestation, there was no difference in the risk of preeclampsia. However, individuals with a good baseline vitamin D level (≥ 30 ng/mL) had fewer adverse outcomes [34]. The lesson from these studies is that timing and baseline status can determine effectiveness. Hu et al. [27] also state that having suitable serum levels of vitamin D in pregnancy could be more important than the dose received.

Evidence for statins, particularly pravastatin, is less conclusive. While pravastatin has been suggested to improve endothelial function and reduce oxidative stress as a component of pathophysiology in preeclampsia, evidence from trials was conflicting. Early randomised trials have been promising in the prevention of early-onset preeclampsia [35], yet meta-analyses were unable to show statistically significant maternal hypertensive results. Although not reaching statistical significance, the trial analysis presented in the study indicated potentially favorable point estimates for selected neonatal outcomes. Pravastatin 20 mg was found to be linked with lower NICU admissions (RR 0.64, 95% CI 0.35–1.16) and neonatal respiratory distress syndrome risk (RR 0.55, 95% CI 0.27–1.12). These results, in conjunction with its SUCRA rankings by relatively high probabilities of neonatal benefit, indicate that pravastatin shows preliminary signals of potential fetal benefit. Large multicenter studies are needed to better understand such trends and to evaluate maternal outcomes. Clinically, this observation can mean that pravastatin might have greater placental or fetal effects than direct maternal effects, potentially by influencing the placental perfusion and anti-inflammatory pathways.

Evidence regarding metformin remains discordant and complex. Certain vitamin D regimens, particularly 4000 IU/day, were associated with a statistically significant estimated risk reduction (RR = 0.21, 95% CI: 0.06–0.72). While metformin did not demonstrate a statistically significant reduction in preeclampsia (RR = 1.98, 95% CI: 0.69–5.73). Although not statistically significant, these findings can represent an array of possible effects, either no effect, an early signal of harm, or explained with confounding by indication, as it is often prescribed to women with higher baseline risks, such as those with obesity or diabetes, rather than a true causal harm. The observed direction of effect may therefore be explained by heterogeneity in baseline risk rather than reflecting a true causal effect. Mechanistic data suggested that metformin may potentially enhance endothelial function and oxidative stress pathways with particular relevance to the pathophysiology of preeclampsia [9], and some systematic reviews report beneficial trends [36–39]. Others are null or inconclusive, perhaps related to heterogeneity of dosing, population, and timing of treatment initiation. An umbrella review in 2024 illustrated that benefits were seen with more intensive dosing regimens [18]. Moreover, some research suggests metformin effects are confined to obese high-risk women, in whom its metabolic effect (i.e., increased insulin sensitivity and weight loss) is most evident [36]. Overall, evidence is suboptimal to recommend routine metformin use for preeclampsia prevention in the general population beyond metabolic indications.

Vitamin D supplementation at 4,000 IU/day showed the most favorable point estimates for preeclampsia and preterm birth within the included trials. High SUCRA rankings suggest a higher probability of a favorable ranking in this network; however, these rankings should be interpreted cautiously and do not confirm clinical superiority, nor do they necessarily correspond to clinically meaningful benefit. Pravastatin may be promising, though evidence remains limited and inconsistent, while metformin shows heterogeneous and inconclusive results. SUCRA rankings should be interpreted cautiously in a clinical context, as they represent probabilistic rankings rather than measures of absolute effect. Moreover, small sample sizes and variable study quality may further limit their interpretability.

These findings appear to be dose-specific and should not be interpreted as a class effect of vitamin D supplementation. Although exploratory SUCRA rankings suggested that vitamin D 60,000 IU/month and 4,000 IU/day had the highest probability of a favorable ranking, the sparse network structure and limited number of informing studies necessitate cautious interpretation.

It is important to interpret some of the statistically significant findings with caution, particularly those that are supported by only a single trial. For instance, the apparent protective effects of vitamin D 60,000 IU/month on preeclampsia, preterm birth, and low birth weight were all derived from Kabuyanga [45] alone. Reliance on a single study limits the robustness and generalizability of these estimates, as they may be influenced by study-specific factors such as design, population characteristics, or risk-of-bias concerns. Consequently, while these findings suggest potential benefit, they should be considered hypothesis-generating rather than confirmatory, highlighting the need for replication in additional well-powered, high-quality trials.

Some statistically significant outcomes in this network meta-analysis were primarily informed by studies assessed as having high or unclear risk of bias. While quantitative sensitivity analyses excluding these studies were not feasible due to the limited number of trials, it is important to acknowledge that the robustness of these effect estimates may be influenced by study-specific biases. Consequently, the findings should be interpreted cautiously and considered hypothesis-generating rather than confirmatory.

Notably, our results differ from those reported by Pérez-López et al. (2015) [33], likely reflecting methodological differences, including the lower and more heterogeneous dosing regimens examined and the reliance on pairwise comparisons, whereas the present network meta-analysis integrates both direct and indirect evidence from higher-dose regimens and more recent randomized trials. Overall, while higher-dose vitamin D appears to be associated with reduced preeclampsia incidence, the evidence for pravastatin and metformin remains inconsistent.

The generalizability of the findings from this network meta-analysis warrants careful consideration. Although all included trials enrolled pregnant women classified as high-risk for preeclampsia, the majority were conducted in limited geographic regions and did not consistently report participant race, ethnicity, or socioeconomic characteristics. As a result, it is unclear to what extent the observed associations apply across diverse racial and ethnic groups, particularly populations known to experience disproportionate burdens of preeclampsia and adverse pregnancy outcomes.

Racial and ethnic differences in baseline risk, vitamin D metabolism, access to prenatal care, and comorbid conditions may modify treatment effects, potentially limiting the external validity of pooled estimates. Variations in healthcare systems, nutritional status, and supplementation practices across settings may influence both effectiveness and safety profiles. Consequently, the results should be interpreted as broadly informative rather than universally applicable, and extrapolation to underrepresented populations should be undertaken with caution. Future trials should prioritize inclusive recruitment and transparent reporting of race and ethnicity to better assess differential treatment effects and improve equity in evidence generation.

Although point estimates suggested a potentially large relative risk reduction with higher-dose vitamin D supplementation, the wide confidence intervals and limited number of contributing trials substantially reduce the precision and certainty of this estimate. However, implementation should consider equity in low-resource regions, where access to supplements may be limited. Potential harms, including vitamin D toxicity, should also be monitored when using higher doses.

For key findings derived from a limited number of studies, the potential for bias warrants careful consideration. For example, the apparent reduction in preeclampsia risk associated with vitamin D 4,000 IU/day (RR = 0.21) was estimated within a sparsely connected network in which contributing trials exhibited heterogeneous risk-of-bias profiles. The indirect nature of this comparison, together with methodological limitations in some primary studies, suggests that the magnitude of this effect should be interpreted with caution and may be particularly sensitive to biases related to non-adherence or incomplete outcome reporting.

Similarly, the observed protective effect of vitamin D 60,000 IU/month against preeclampsia and preterm birth is primarily informed by a single large randomized trial. Although this study demonstrated relatively precise and consistent estimates across neonatal outcomes, it was assessed as having some concerns regarding risk of bias. Consequently, the reliance on a single source of evidence underscores the need for replication in future, methodologically rigorous, randomized controlled trials.

For pravastatin, where the evidence base is limited to two trials, one judged to have some concerns and the other a high risk of bias, the non-significant trends observed for outcomes such as NICU admission and respiratory distress syndrome should be regarded as preliminary. Potential performance and detection biases in these studies imply that the true effect may be either overestimated or underestimated.

Importantly, the observed associations may reflect differences in underlying population characteristics rather than true differences in treatment efficacy, given the limited ability to adjust for effect modifiers in study-level analyses. Variation in gestational age at treatment initiation represents a potential effect modifier; however, sensitivity or stratified analyses by initiation timing were not feasible due to inconsistent reporting and overlapping initiation windows across trials. Consequently, estimates, particularly for statins, should be interpreted with caution.

Overall, while this network meta-analysis provides comparative estimates across interventions, the findings—particularly for dose-specific vitamin D and pravastatin should be interpreted in the context of the limited, heterogeneous, and sparse evidence base. The observed effect estimates may be uncertain and potentially unstable due to study-level heterogeneity and risk-of-bias concerns. Accordingly, any apparent signal, especially in favor of vitamin D, should be interpreted cautiously to avoid overinterpretation. These results should be considered hypothesis-generating and underscore the need for well-designed, adequately powered, head-to-head randomized trials to confirm these associations.Current evidence does not support replacing established prophylactic strategies.

## Limitations

Our study has some limitations. The restriction of the search window (2020–2025) and the exclusion of trials involving aspirin in the comparator arm might limit the generalizability of our findings to all clinical settings.

Clinical heterogeneity across included trials represents a key limitation. The definition of "high-risk pregnancy" was not standardised across studies. Some trials enrolled women based on obstetric history such as prior preeclampsia, while others included women solely on the basis of vitamin D deficiency or metabolic comorbidities. Dosing regimens, timing of supplementation, baseline vitamin D status, duration of intervention, and underlying maternal risk profiles further varied across trials, threatening the transitivity assumption and limiting the comparability of pooled estimates.

Direct head-to-head comparisons were very few, and as a result, several estimates relied heavily on indirect evidence. Treatment rankings derived from SUCRA values should therefore be interpreted with caution, as they may be unstable in small and weakly connected networks.

The number of trials evaluating individual interventions was particularly limited. For metformin and pravastatin, evidence was based on a small number of studies, and several comparisons were informed by a single trial. Due to these small sample sizes, sparse networks, and few low-risk-of-bias studies, treatment effect estimates and ranking probabilities should be interpreted with caution, and findings should not inform current clinical decision-making.

Some included studies [41, 43] lacked baseline data for the control group, and others had small sample sizes. These limitations may affect the reliability and precision of our findings and should be considered when interpreting the results.

The timing of intervention initiation varied widely and was inconsistently reported across studies, precluding sensitivity analyses excluding late initiation or stratification by gestational age; observed effects may therefore reflect differences in disease stage rather than true intervention efficacy.

There was also an imbalance in participant allocation (2281 in intervention arms vs. 1741 in control arms), with unexplained attrition (~ 56 participants), raising concerns regarding potential selection and attrition bias.

Planned subgroup analyses stratified by baseline vitamin D status, obesity, diabetes, or insulin resistance were not feasible due to insufficient and inconsistent reporting across trials. Consequently, potential effect modification by metabolic risk status, particularly relevant for metformin, could not be explored.

Dose–response relationships for vitamin D could not be formally evaluated using network meta‑regression or dose–response models because of the limited number of trials and the sparse network structure, highlighting an important area for future research. Vitamin D should therefore not be considered a class effect, as dosing regimens varied substantially across included trials. Although higher doses appeared to rank more favorably, these findings should be interpreted as hypothesis‑generating rather than practice‑changing.

Although trials with aspirin in the comparator arm were excluded to minimize co‑intervention bias, incomplete reporting of background care in some studies means that unmeasured co‑interventions cannot be entirely ruled out. Given that low-dose aspirin represents standard prophylaxis in many high-risk pregnancies, the exclusion of trials including aspirin in comparator arms may limit applicability of findings to real-world clinical practice, where combination strategies are common.

The evidence base was limited to randomized trials published in English, which may have excluded relevant studies conducted in other languages and introduced language bias. In addition, gray literature and unpublished trials were not systematically included, raising the possibility of publication bias, particularly in the context of a small and fragmented evidence base.

Publication bias cannot be excluded, as the small number of trials per comparison precluded formal assessment using funnel plots or statistical tests.

Taken together, these limitations highlight the need for cautious interpretation. In line with recent systematic reviews in women’s health, the results were interpreted with particular caution due to the sparse network, reliance on indirect evidence, and clinical heterogeneity, reflecting methodological considerations relevant to meta-analytic synthesis and the design of preventive or supportive interventions [40].

## Conclusion

This network meta-analysis provides comparative estimates suggesting possible associations between higher-dose vitamin D regimens and reduced probabilities of certain adverse outcomes; however, these findings are based on sparse and heterogeneous evidence and should not be interpreted as proof of clinical effectiveness. This network meta-analysis suggests that higher-dose vitamin D (60,000 IU/month and 4000 IU/day) was associated with patterns consistent with lower estimated probabilities of preeclampsia and related adverse outcomes, including outcomes such as preterm birth and low birth weight, among high-risk pregnancies. These results reflect comparative associations rather than established clinical effectiveness. Estimates for pravastatin suggested numerical trends toward differences in neonatal outcomes, while metformin showed lower probabilistic rankings; however, these findings were imprecise and not statistically significant.

It is important to emphasize that treatment rankings derived from this network meta-analysis are theoretical and probabilistic, based on SUCRA values, and should not be interpreted as clinical recommendations. They provide a comparative estimate of potential associations rather than definitive guidance for practice.

Although vitamin D consistently showed comparatively higher probabilistic rankings across several outcomes, these observations must be interpreted cautiously, as most contributing studies were assessed as having moderate or high risk of bias. Future large-scale, well-designed randomized controlled trials are needed to clarify optimal interventions, dosing, and timing. Until such evidence is available, these findings serve primarily to generate hypotheses and inform future research rather than to guide clinical practice. This network meta-analysis provides a comparative overview of pharmacological interventions that may reduce the risk of preeclampsia; however, the available evidence is limited and the network structure is sparse. Although some interventions, including certain vitamin D regimens, showed potentially favorable estimates, these findings are uncertain and largely based on indirect evidence. Therefore, the results should be considered hypothesis-generating rather than practice-changing. Future large-scale, well-designed randomized controlled trials with standardized populations and dosing regimens are needed to provide more definitive comparative evidence.

## Supplementary Information

Additional file 1.

Additional file 2.

Additional file 3.

Additional file 4.

Additional file 5.

## Data Availability

All data generated or analyzed during this study are included in this published article.

## Abbreviations

RCTs: Randomized Controlled Trials
NICU: Neonatal Intensive Care Unit
RDS: Respiratory Distress Syndrome
RRs: Risk Ratios
CIs: Confidence Intervals
SUCRA: Surface Under the Cumulative Ranking Curve
IU: International Units
sFlt-1: Soluble Fms-like Tyrosine Kinase-1
PlGF: Placental Growth Factor
VEGF: Vascular Endothelial Growth Factor
PRISMA: Preferred Reporting Items for Systematic Reviews and Meta-Analyses
SGA: Small for Gestational Age
IUGR: Intrauterine Growth Restriction
HMG-CoA: 3-Hydroxy-3-Methylglutaryl–Coenzyme A
EPA: Eicosapentaenoic Acid
DHA: Docosahexaenoic Acid
BMI: Body Mass Index
SD: Standard deviation
RoB: Risk of Bias
I^2^: I-squared statistic
τ^2^: Tau-squared
LBW: Low Birth Weight
PICOS: Population, Intervention, Comparison, Outcome, Study design
OR: Odds Ratio
