# Supplementary material for: Comparative effectiveness of statins, metformin, and vitamin D, for prevention of preeclampsia: a systematic review and network meta-analysis of randomized trials
Source: BMC Pregnancy Childbirth. 2026 Oct 2;26:1101. doi: 10.1186/s12884-026-09920-6 (PMC13632318; doi:10.1186/s12884-026-09920-6)
Supplement: Supplementary file 1 [file 12884_2026_9920_MOESM2_ESM.pdf]

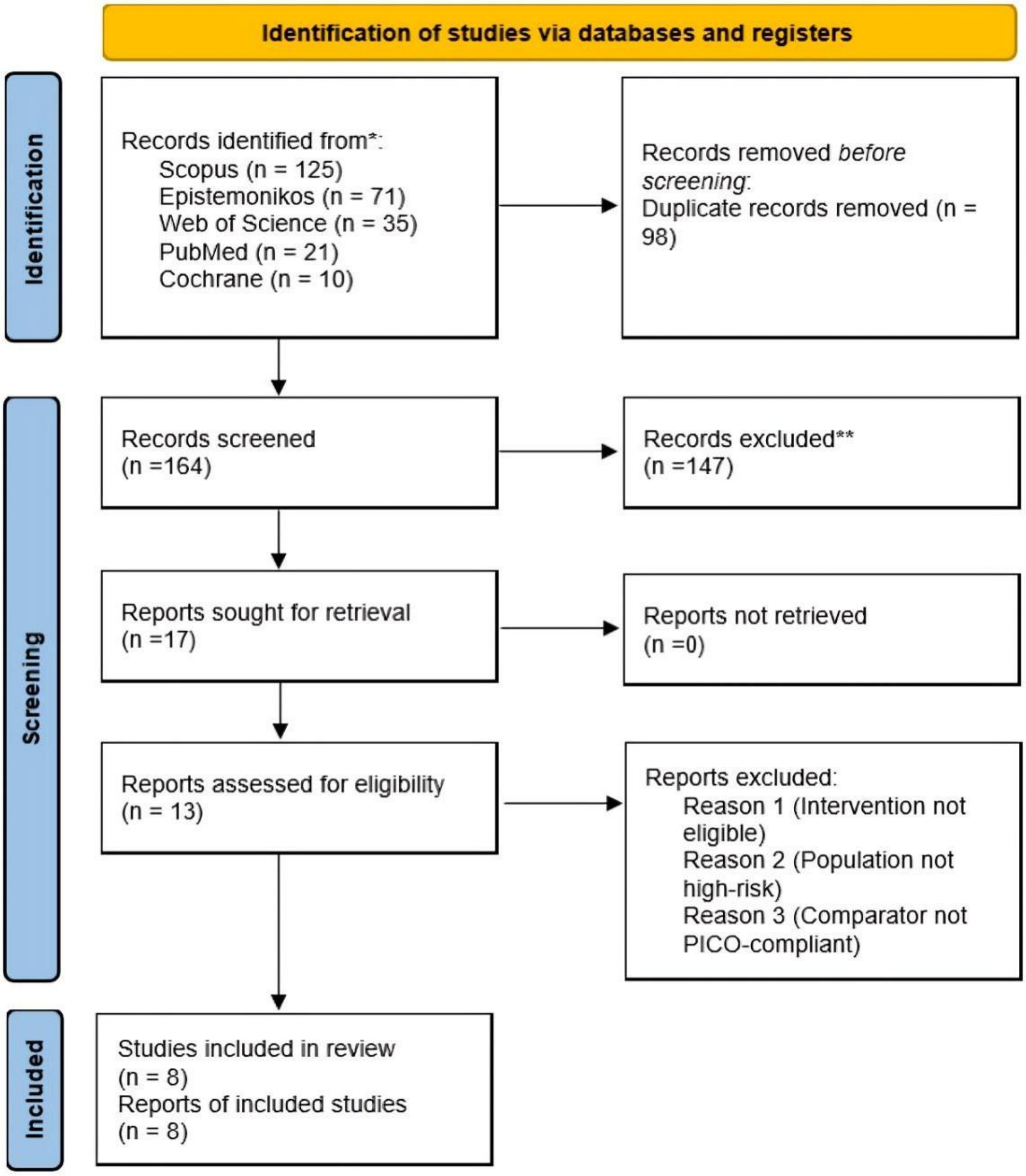

Figure 1 shows the PRISMA flowchart of the included studies

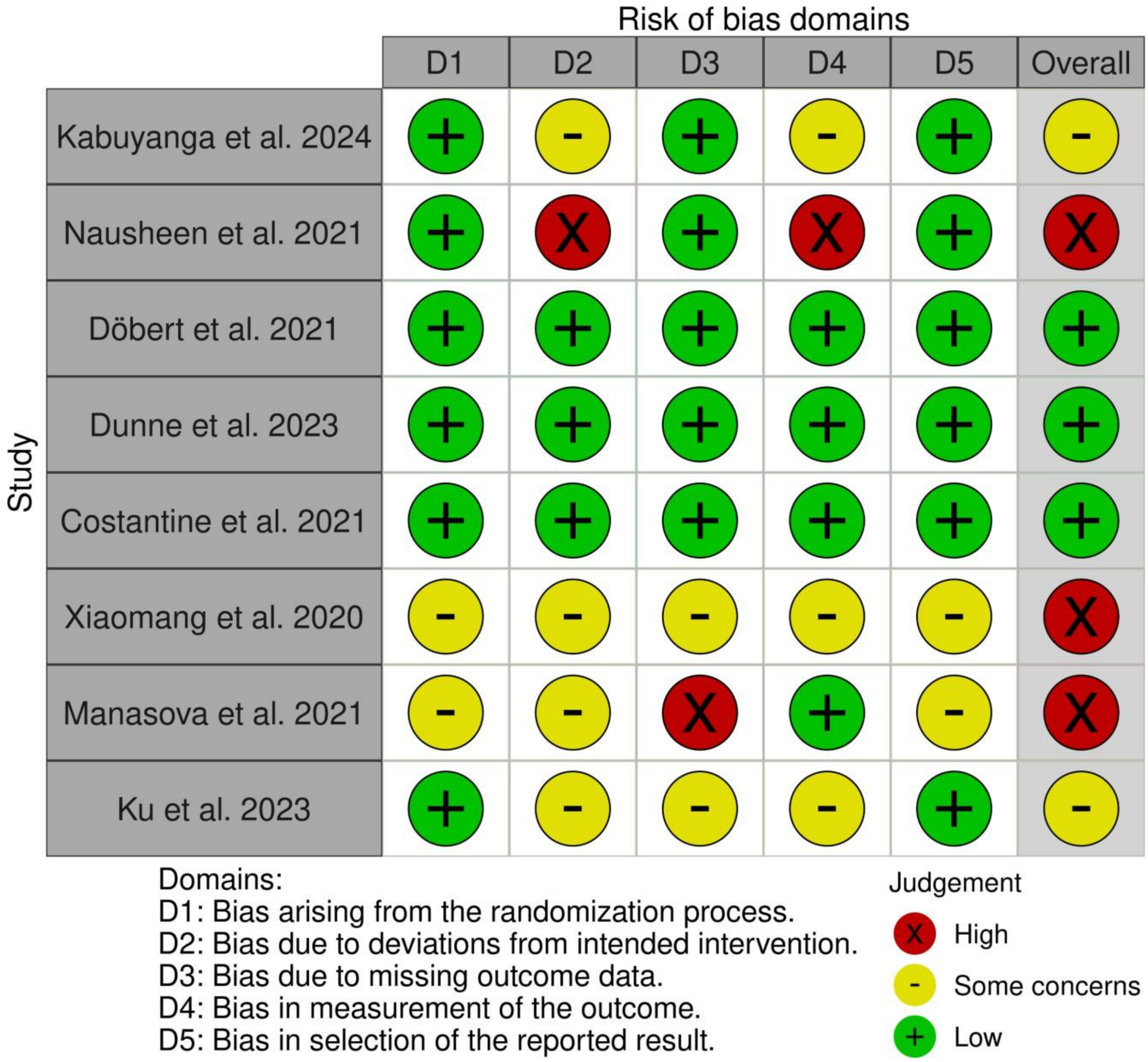

Figure 2 shows Risk of bias assessment of included randomized controlled trials using the Cochrane Risk of Bias 2 tool. Domains assessed include randomization process, deviations from intended interventions, missing outcome data, outcome measurement, and selective reporting. Green symbols indicate low risk of bias, yellow symbols indicate some concerns, and red symbols indicate high risk of bias.

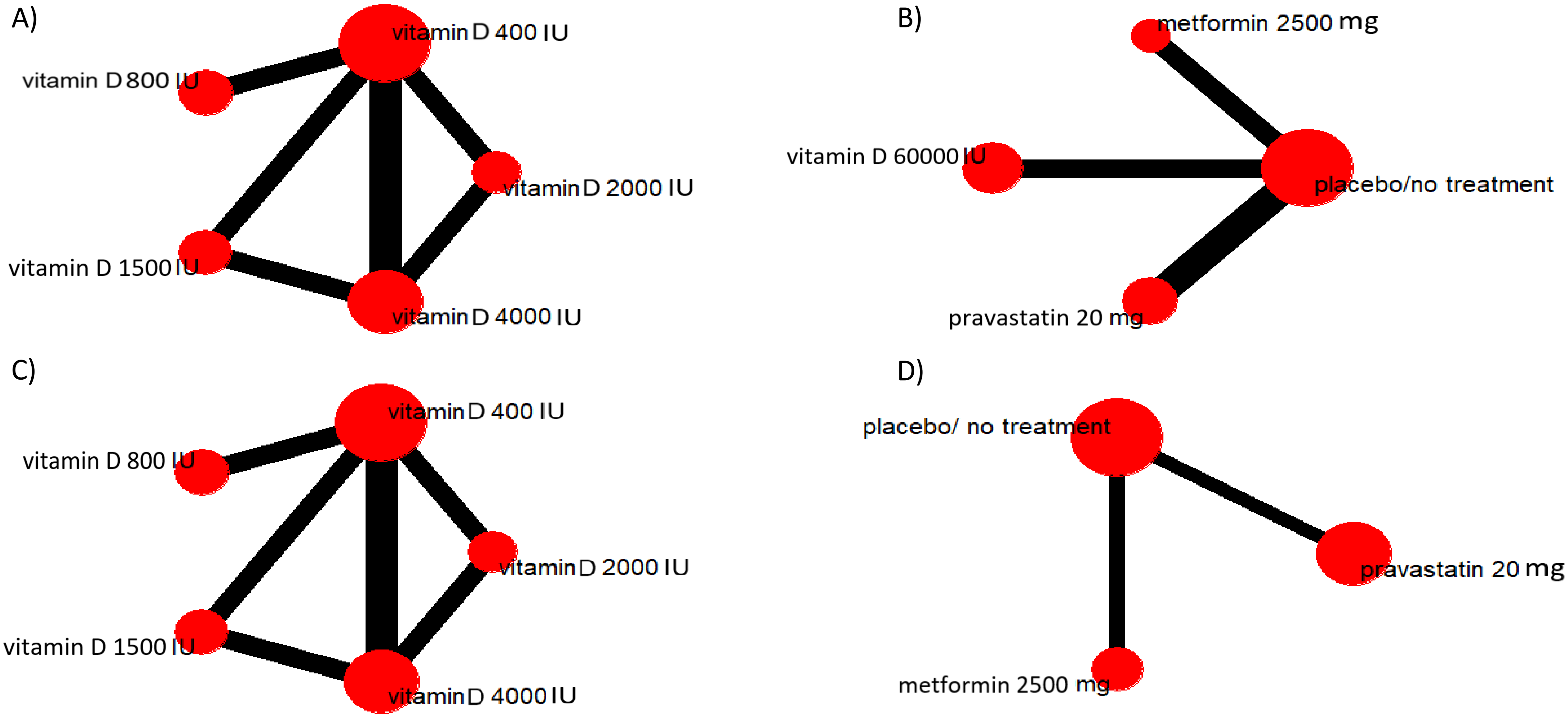

Figure 3. Network plots illustrating the structure of the treatment networks for maternal and neonatal outcomes. Nodes represent interventions, and node size is proportional to the number of randomized participants. Edges represent direct comparisons, with edge thickness corresponding to the number of trials contributing to each comparison. Comparisons without a connecting edge are informed exclusively by indirect evidence. (A) Pre-eclampsia (vitamin D 400 IU as comparator); (B) Pre-eclampsia (placebo as comparator); (C) Gestational diabetes; (D) Gestational hypertension.

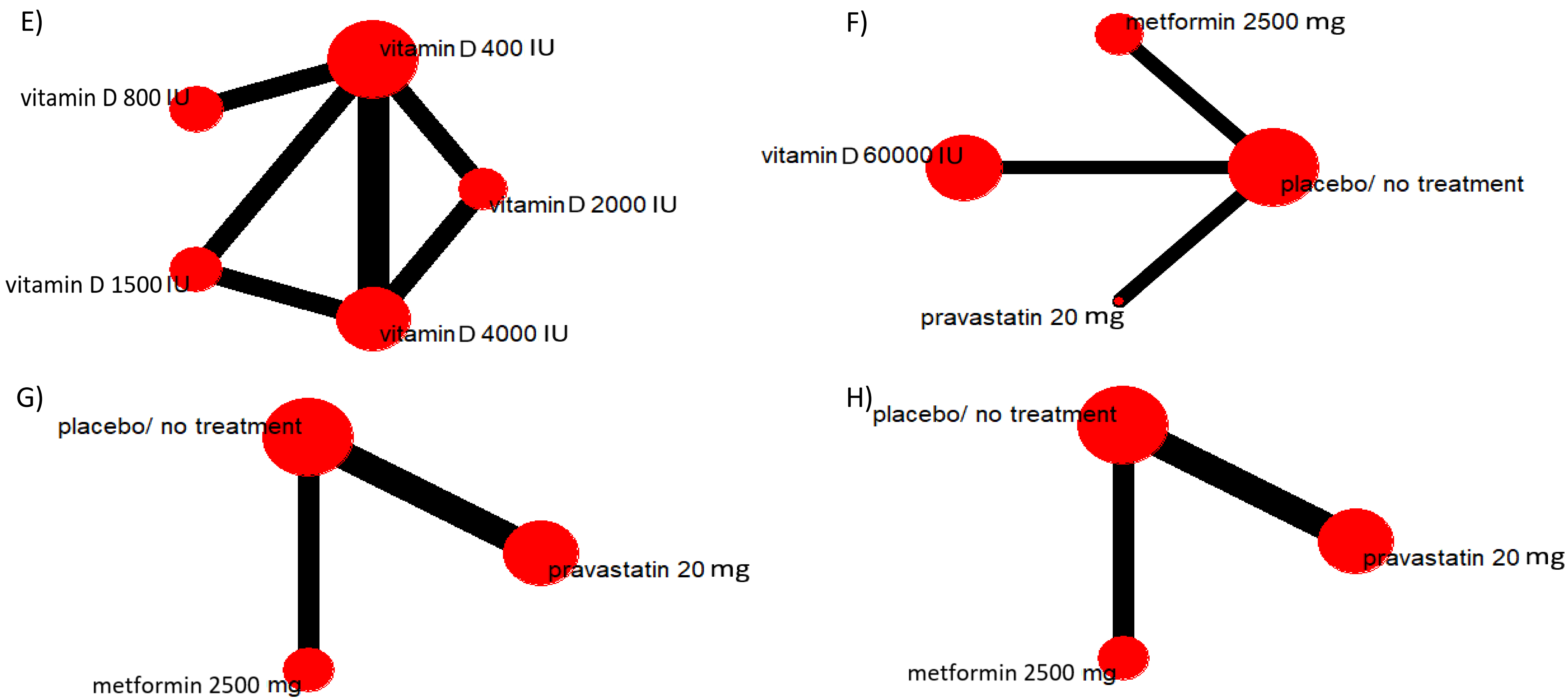

Figure 3 (Continued) shows network plots illustrating the structure of the treatment networks for maternal and neonatal outcomes. Nodes represent interventions, and node size is proportional to the number of randomized participants. Edges represent direct comparisons, with edge thickness corresponding to the number of trials contributing to each comparison. Comparisons without a connecting edge are informed exclusively by indirect evidence. (E) Preterm birth (vitamin D 400 IU as comparator); (F) Preterm birth (placebo as comparator); (G) NICU admission; (H) Respiratory distress syndrome (RDS).

I)

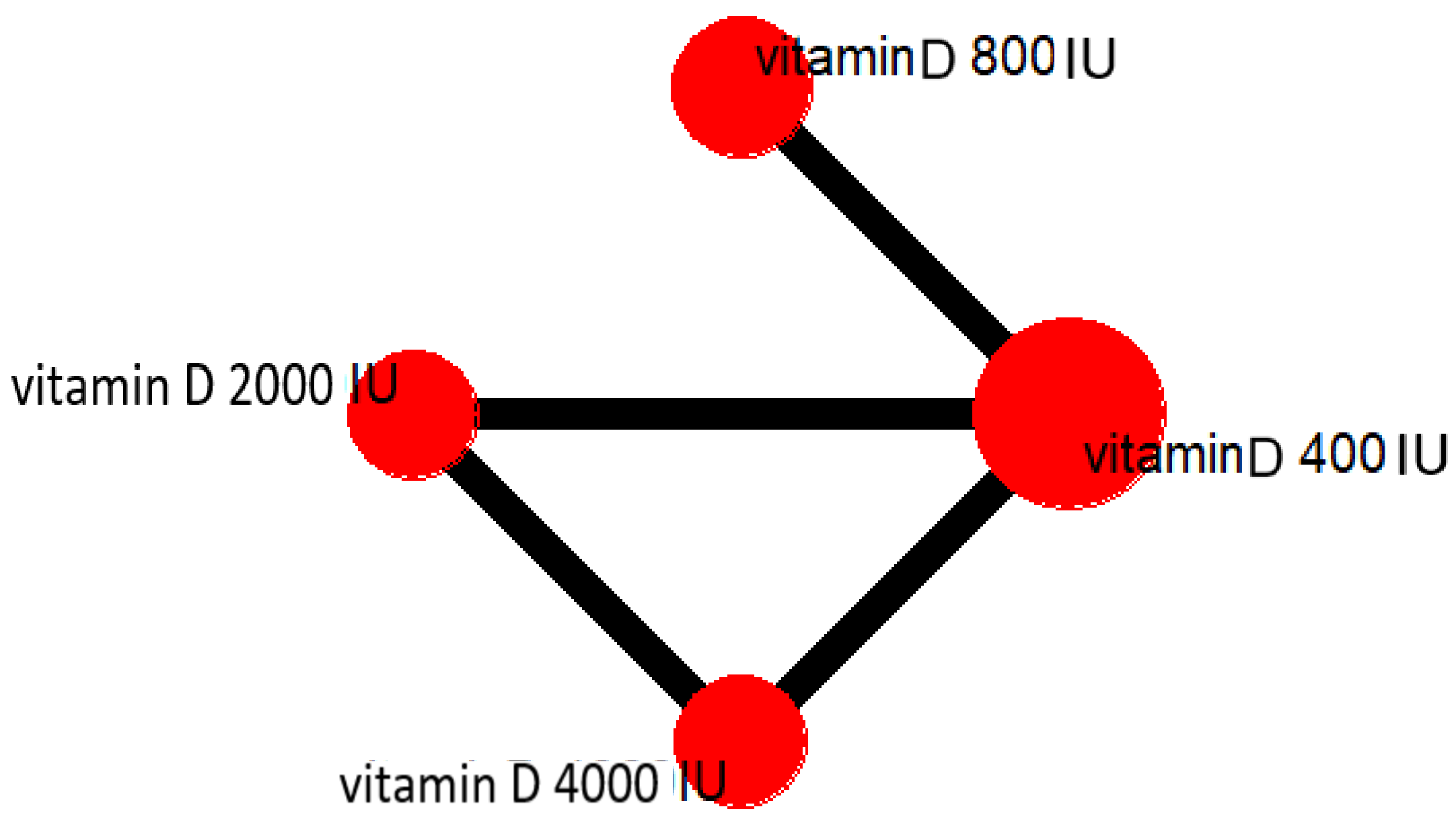

J)

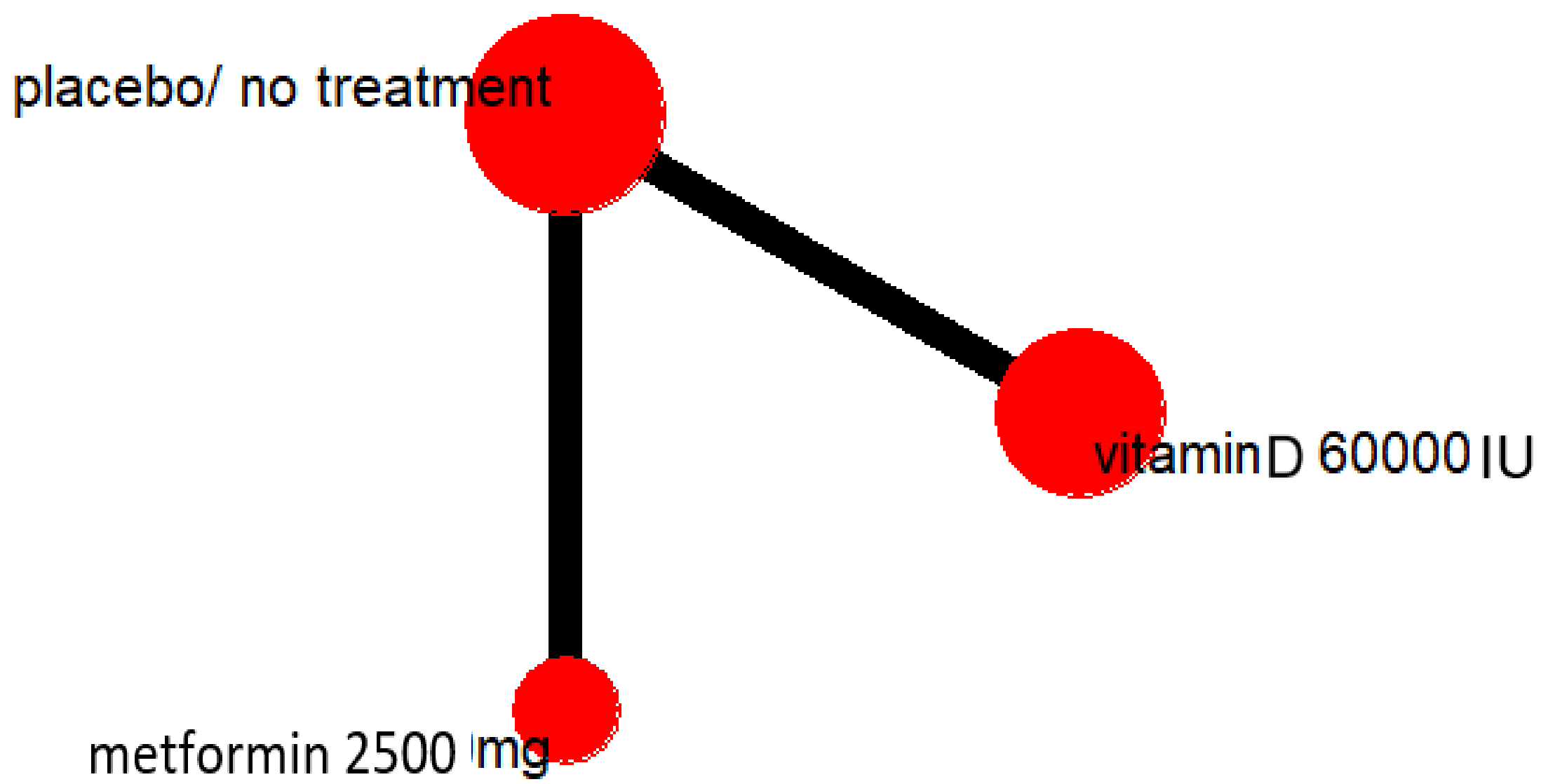

Figure 3 (Continued) shows network plots illustrating the structure of the treatment networks for maternal and neonatal outcomes. Nodes represent interventions, and node size is proportional to the number of randomized participants. Edges represent direct comparisons, with edge thickness corresponding to the number of trials contributing to each comparison. Comparisons without a connecting edge are informed exclusively by indirect evidence. (I) Low birth weight (vitamin D 400 IU as comparator); (J) Low birth weight (placebo as comparator).

A)

vitamin D 1500 IU	.	1.09 (0.58 to 2.08)	1.07 (0.56 to 2.03)	.
0.45 (0.12 to 1.63)	vitamin D 2000 IU	2.09 (0.53 to 8.15)	3.00 (0.62 to 14.55)	.
1.07 (0.57 to 2.01)	2.39 (0.73 to 7.86)	vitamin D 4000 IU	1.02 (0.56 to 1.89)	.
1.10 (0.58 to 2.06)	2.45 (0.72 to 8.30)	1.02 (0.56 to 1.89)	vitamin D 400 IU	0.92 (0.55 to 1.54)
1.01 (0.45 to 2.27)	2.25 (0.60 to 8.47)	0.94 (0.42 to 2.09)	0.92 (0.55 to 1.54)	vitamin D 800 IU

B)

metformin 2500 mg	1.98 (0.69 to 5.73)	.	.
1.98 (0.69 to 5.73)	placepo/ no treatment	0.97 (0.73 to 1.30)	2.76 (1.44 to 5.29)
1.93 (0.64 to 5.79)	0.97 (0.73 to 1.30)	pravastatin 20 mg	.
5.48 (1.58 to 19.00)	2.76 (1.44 to 5.29)	2.83 (1.39 to 5.77)	vitamin D 60000 IU

C)

vitamin D 1500 IU	.	1.09 (0.58 to 2.08)	1.07 (0.56 to 2.03)	.
0.45 (0.12 to 1.63)	vitamin D 2000 IU	2.09 (0.53 to 8.15)	3.00 (0.62 tp 14.55)	.
1.07 (0.57 to 2.01)	2.39 (0.73 to 7.86)	vitamin D 4000 IU	1.02 (0.56 to 1.89)	.
1.10 (0.58 to 2.06)	2.45 (0.72 to 8.30)	1.02 (0.56 to 1.89)	vitamin D 400 IU	0.92 (0.55 to 1.54)
1.01 (0.45 to 2.27)	2.25 (0.60 to 8.47)	0.94 (0.42 to 2.09)	0.92 (0.55 to 1.54)	vitamin D 800 IU

D)

metformin 2500 mg	1.10 (0.68 to 1.78)	.
1.10 (0.68 to 1.78)	placebo/ no treatment	0.91 (0.70 to 1.18)
1.00 (0.58 to 1.72)	0.91 (0.70 to 1.18)	pravastatin 20 mg

E)

vitamin D 1500 IU	.	0.98 (0.57 to 1.70)	1.06 (0.6 to 1.86)	.
1.35 (0.69 to 2.63)	vitamin D 2000 IU	0.91 (0.53 to 1.56)	0.65 (0.39 to 1.06)	.
1.10 (0.66 to 1.84)	0.82 (0.49 to 1.36)	vitamin D 4000 IU	0.85 (0.59 to 1.21)	.
0.93 (0.56 to 1.56)	0.69 (0.43 to 1.12)	0.85 (0.59 to 1.21)	vitamin D 400 IU	0.62 (0.21 to 1.86)
0.58 (0.17 to 1.95)	0.43 (1.13 to 1.43)	0.53 (0.17 to 1.67)	0.62 (0.21 to 1.86)	vitamin D 800 IU

F)

metformin 2500 mg	1.40 (0.77 to 2.55)	.	.
1.40 (0.77 to 2.55)	placebo/ no treatment	2.00 (0.68 to 5.85)	1.97 (1.27 to 3.06)
2.08 (0.82 to 9.57)	2.00 (0.68 to 5.85)	pravastatin 20 mg	.
2.76 (1.31 to 5.80)	1.97 (1.27 to 3.06)	0.99 (0.31 to 3.15)	vitamin D 60000 IU

G)

metformin 2500 mg	1.23 (0.81 to 1.89)	.
1.23 (0.8 to 1.89)	placebo/ no treatment	1.57 (0.86 to 2.83)
1.93 (0.93 to 4.00)	1.57 (0.86 to 2.83)	pravastatin 20 mg

Figure 4. League tables and network meta-analysis estimates for maternal and neonatal outcomes. Each table is presented with the specified reference comparator as indicated in the panel title. Estimates are derived from direct, indirect, or mixed evidence depending on data availability within each network. Where both direct and indirect evidence were available, mixed network estimates are presented. (A) Pre-eclampsia (vitamin D 400 IU as comparator); (B) Pre- eclampsia (placebo as comparator); (C) Gestational diabetes; (D) Gestational hypertension; (E) Preterm birth (vitamin D 400 IU as comparator); (F) Preterm birth (placebo as comparator); (G) NICU admission.

H)

metformin 2500 mg	1.32 (0.74 to 2.38)	.
1.32 (0.74 to 2.38)	placebo/ no treatment	1.83 (0.89 to 3.74)
2.42 (0.96 to 6.10)	1.83 (0.89 to 3.74)	pravastatin 20 mg

I)

vitamin D 2000 IU	1.42 (0.75 to 2.69)	1.27 (0.68 to 2.37)	.
1.42 (0.75 to 2.69)	vitamin D 4000 IU	0.89 (0.45 to 1.77)	.
1.27 (0.68 to 2.37)	0.89 (0.45 to 1.77)	vitamin D 400 IU	0.78 (0.30 to 2.03)
0.99 (0.31 to 3.10)	0.70 (0.21 to 2.26)	0.78 (0.30 to 2.03)	vitamin D 800 IU

J)

metformin 2500 mg	1.76 (0.79 to 3.92)	.
1.76 (0.79 to 3.92)	placebo/ no treatment	2.31 (1.48 to 3.62)
4.08 (1.64 to 10.20)	2.31 (1.48 to 3.62)	vitamin D 60000 IU

Figure 4 (Continued) shows league tables and network meta-analysis estimates for maternal and neonatal outcomes. Each table is presented with the specified reference comparator as indicated in the panel title. Estimates are derived from direct, indirect, or mixed evidence depending on data availability within each network. Where both direct and indirect evidence were available, mixed network estimates are presented: (H) Respiratory distress syndrome (RDS); (I) Low birth weight (vitamin D 400 IU as comparator); (J) Low birth weight (placebo as comparator).

A)

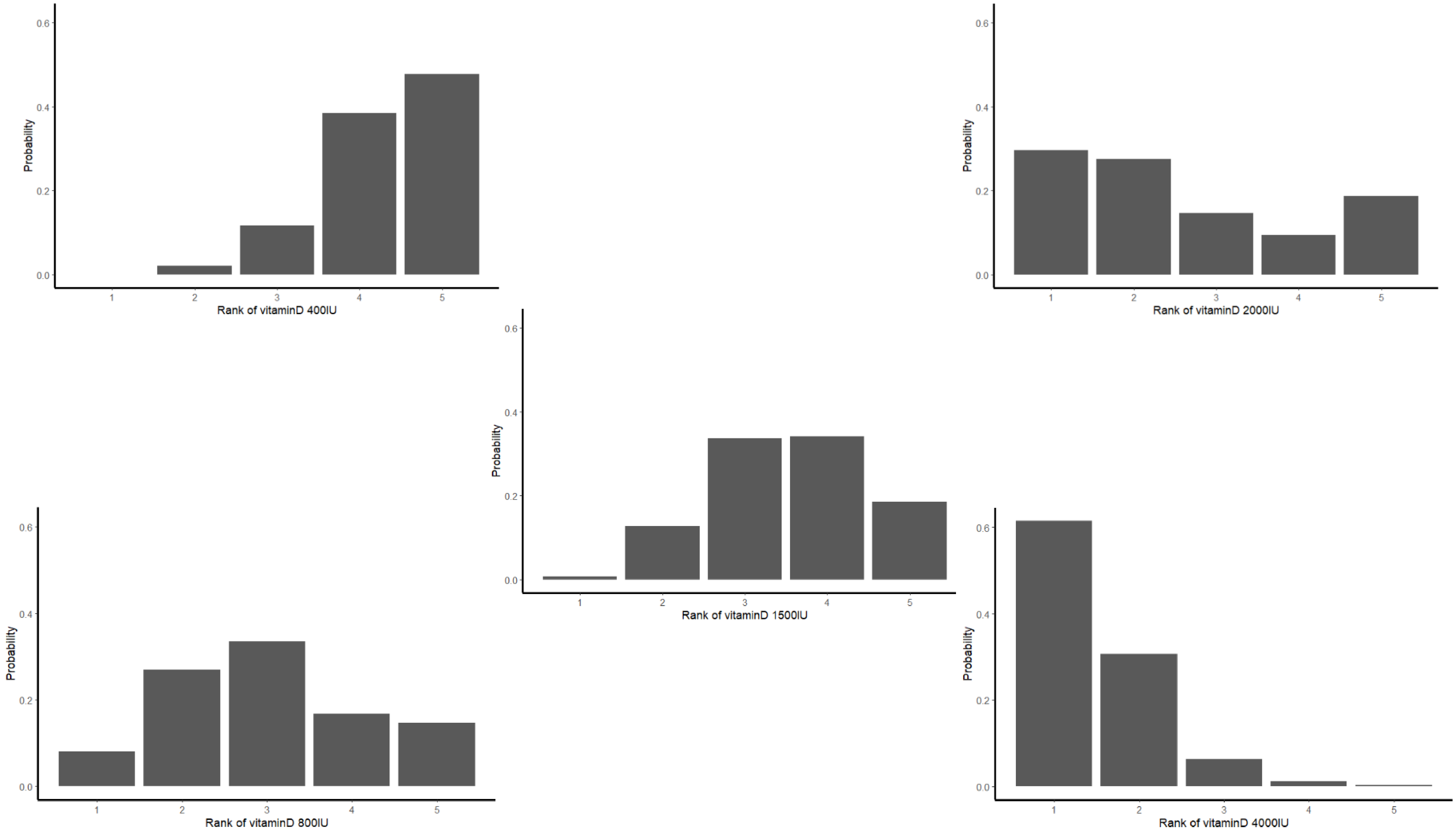

Figure 5 shows Rankograms of mother outcomes: (A) Pre-eclampsia (vitamin D 400IU as comparator)

B)

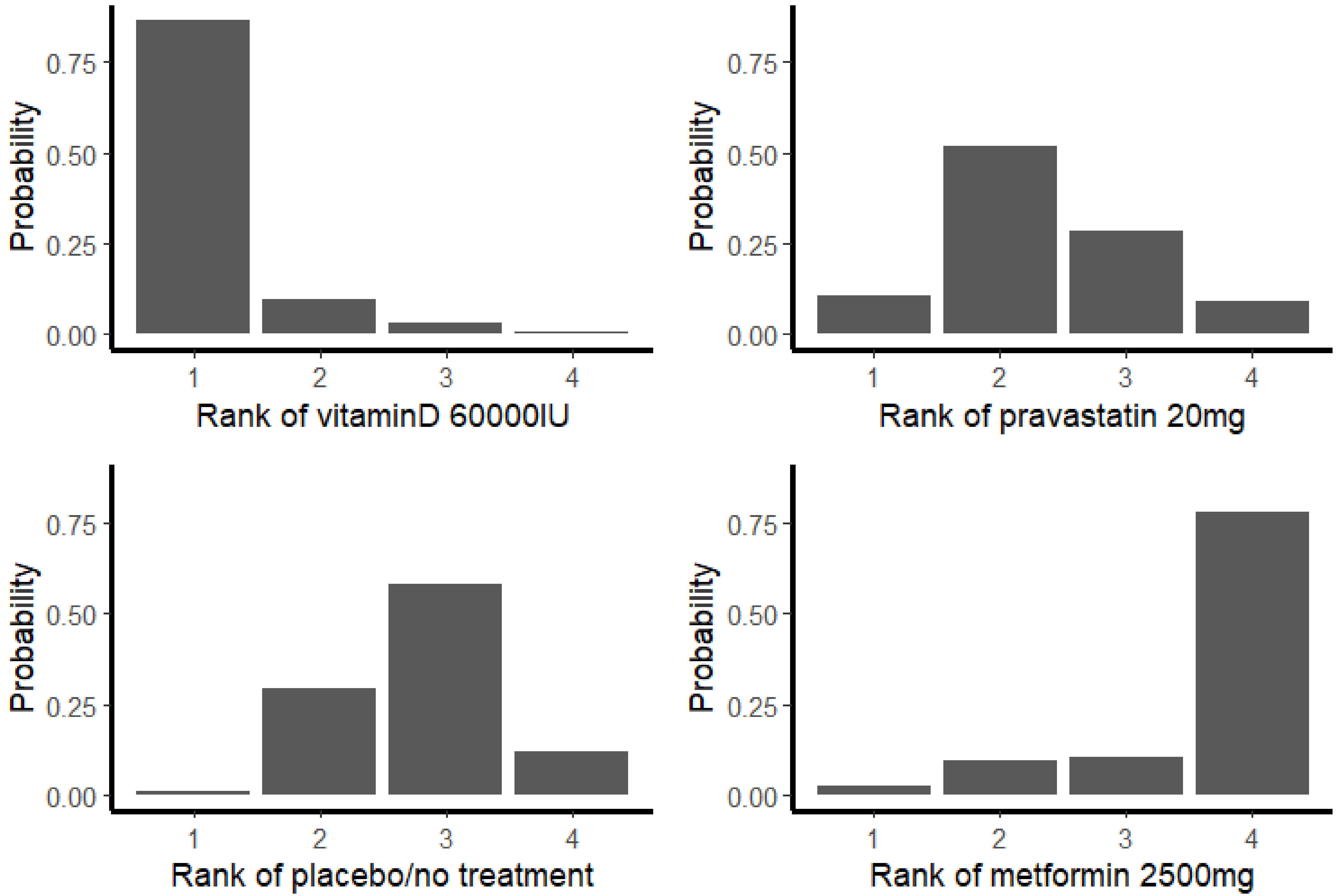

Figure 5 “Continue” shows Rankograms of mother outcomes: (B) Pre-eclampsia (placebo as comparator)

C)

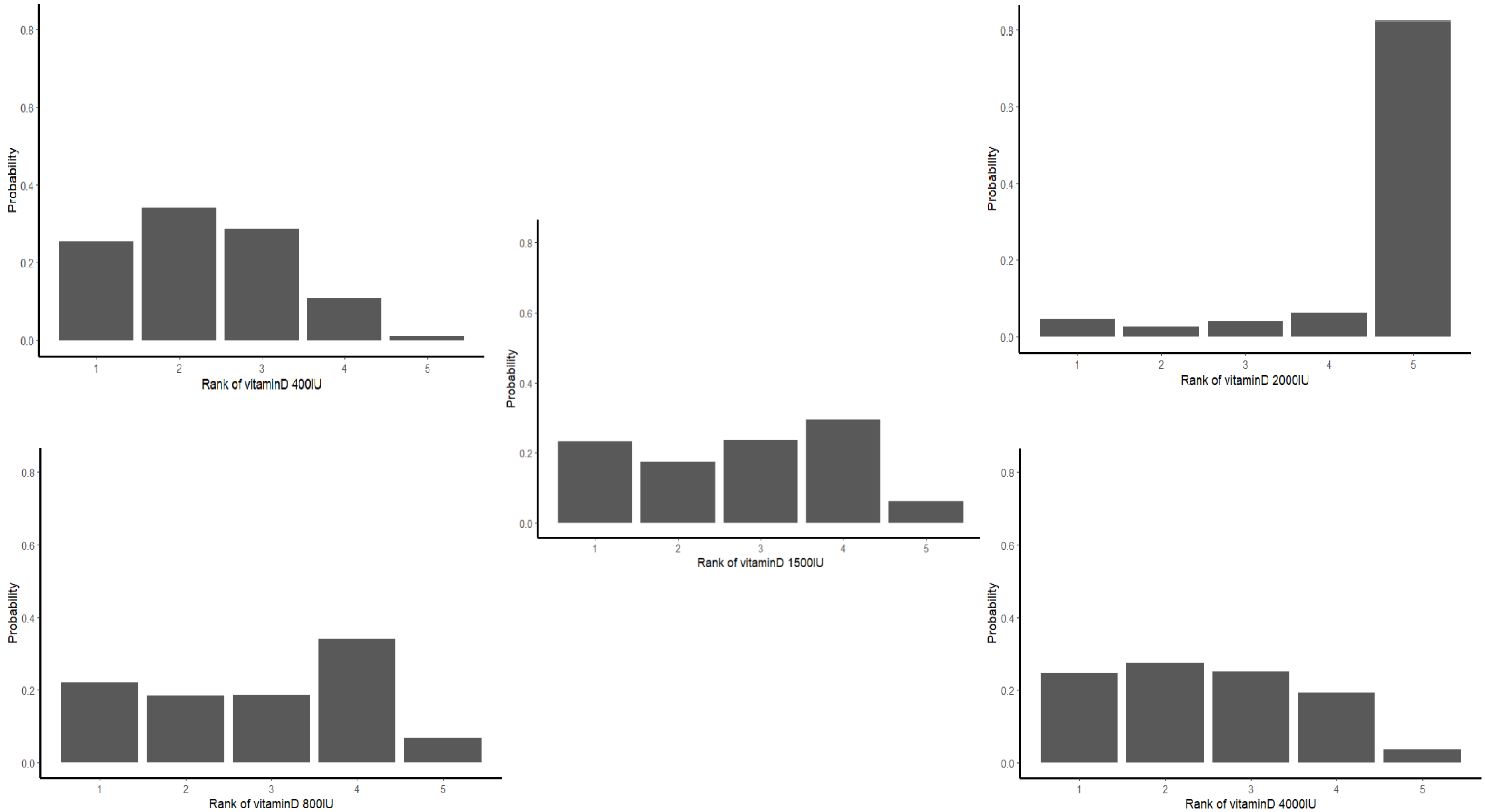

Figure 5 “Continue” shows Rankograms of mother outcomes: (C) Gestational diabetes

D)

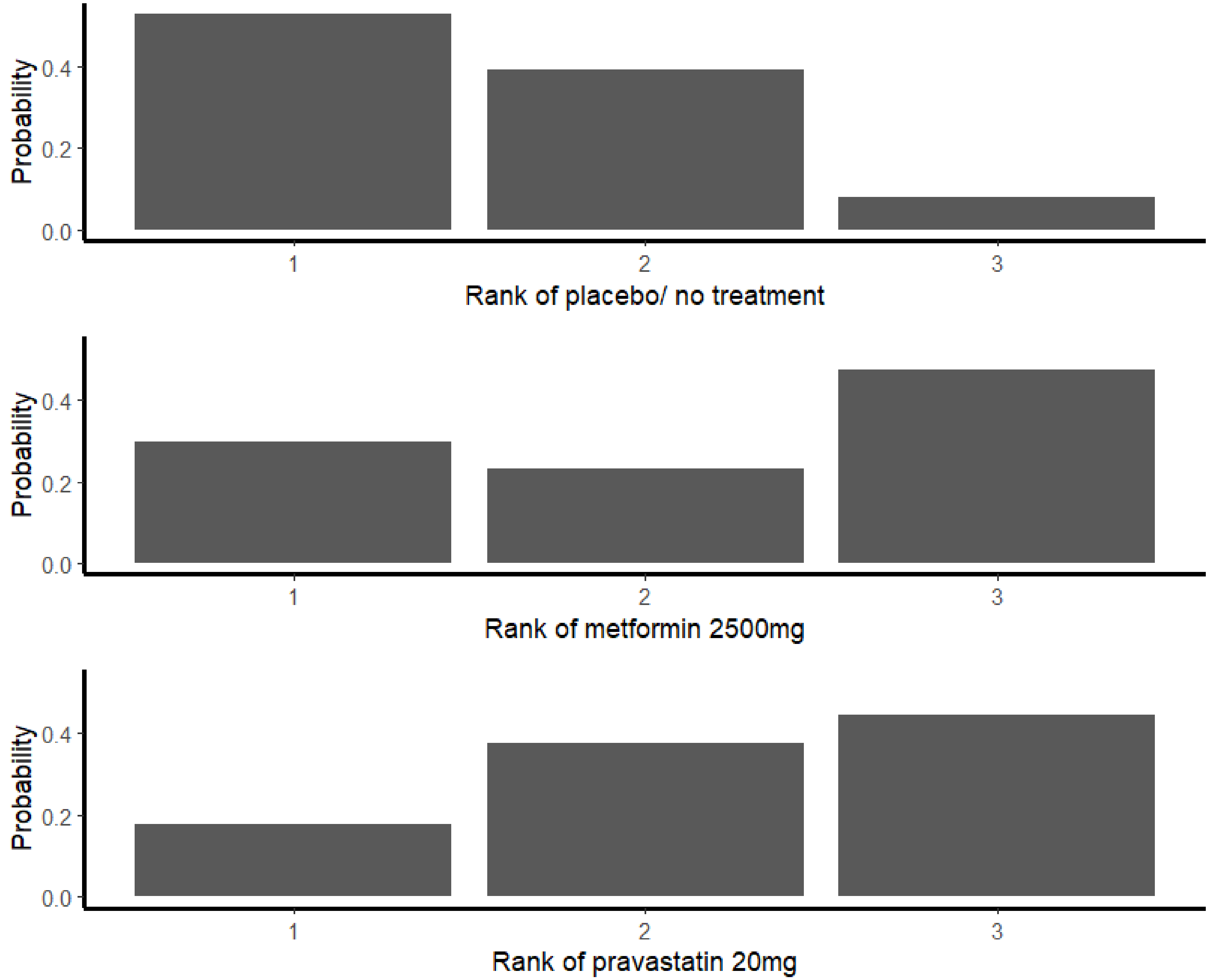

Figure 5 “Continue” shows Rankograms of mother outcomes: (D) Gestational hypertension

A)

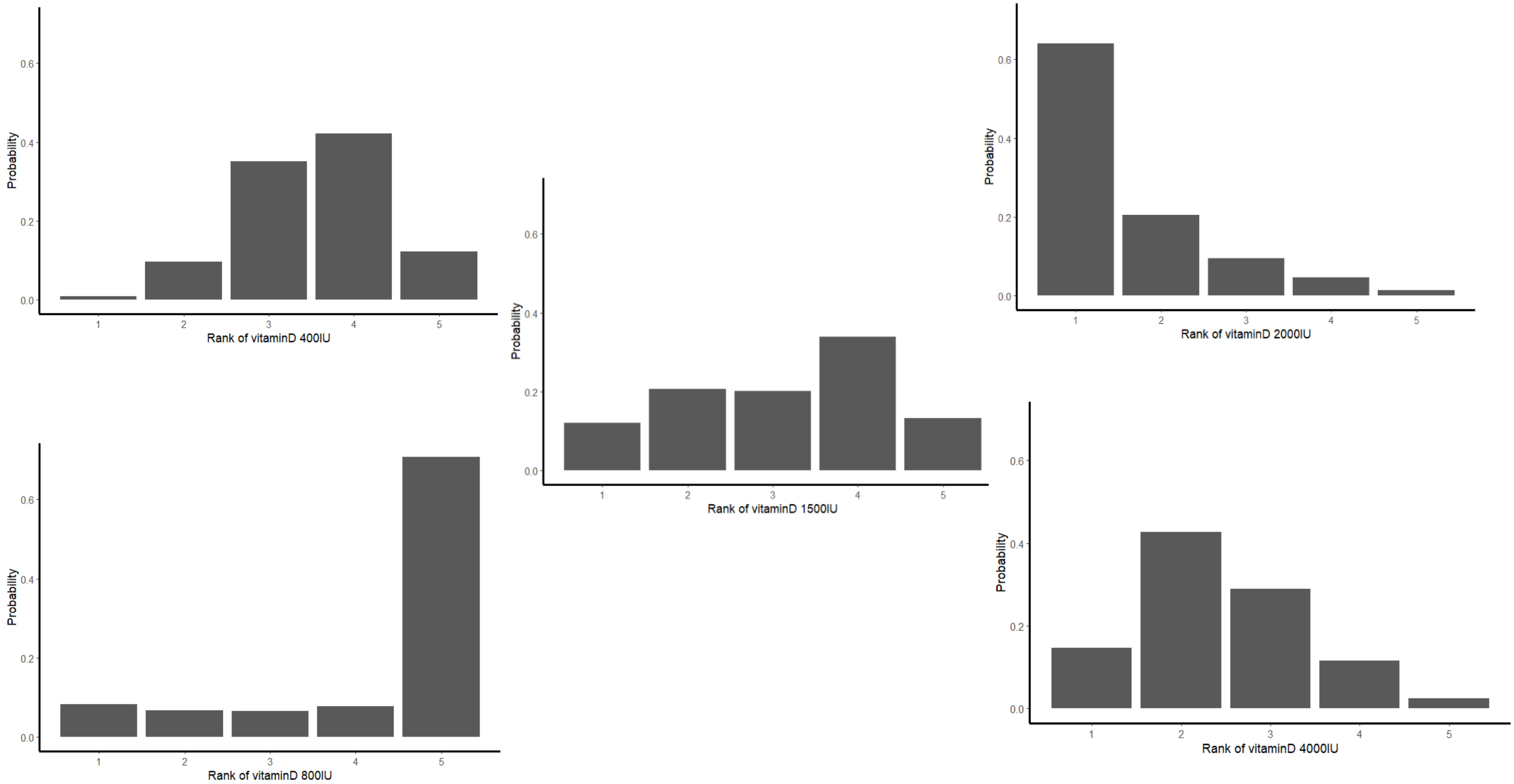

Figure 6 shows Rankograms of neonates outcomes: (A) Pre-term birth (vitamin D 400IU as comparator)

B)

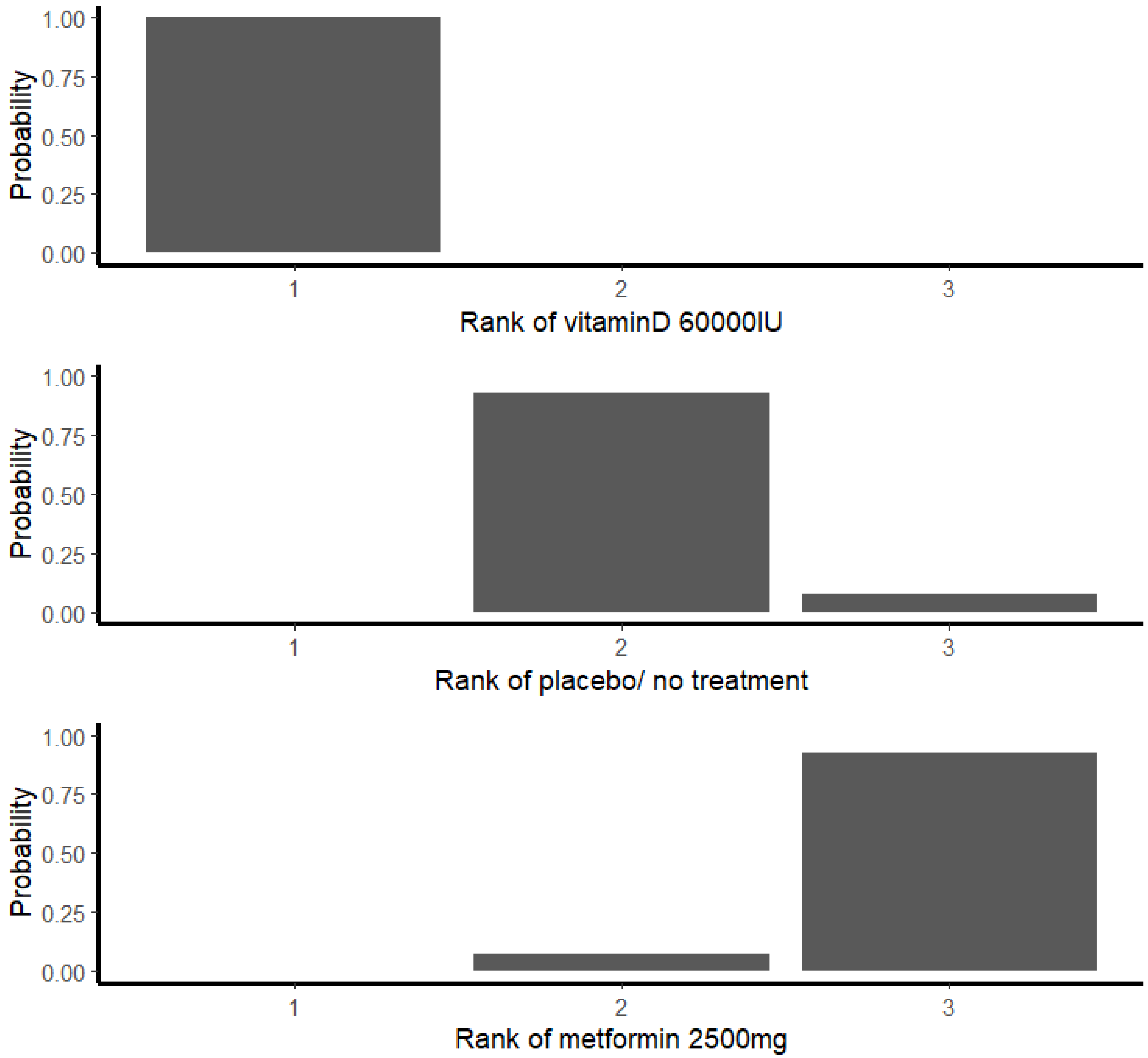

Figure 6 “Continue” shows Rankograms of neonates outcomes: (B) Pre-term birth (placebo as comparator)

C)

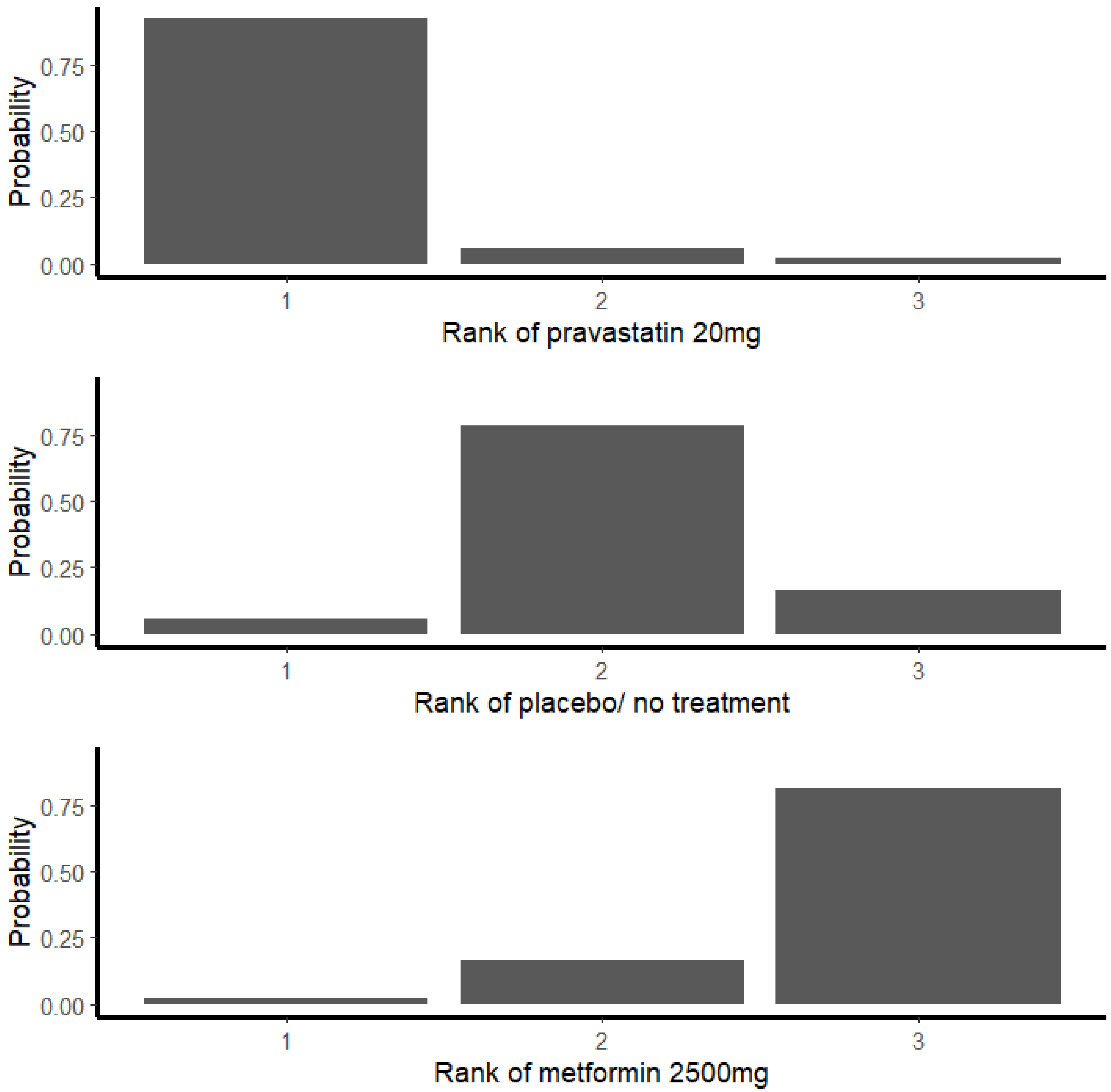

Figure 6 “Continue” shows Rankograms of neonates outcomes: (C) NICU admission

D)

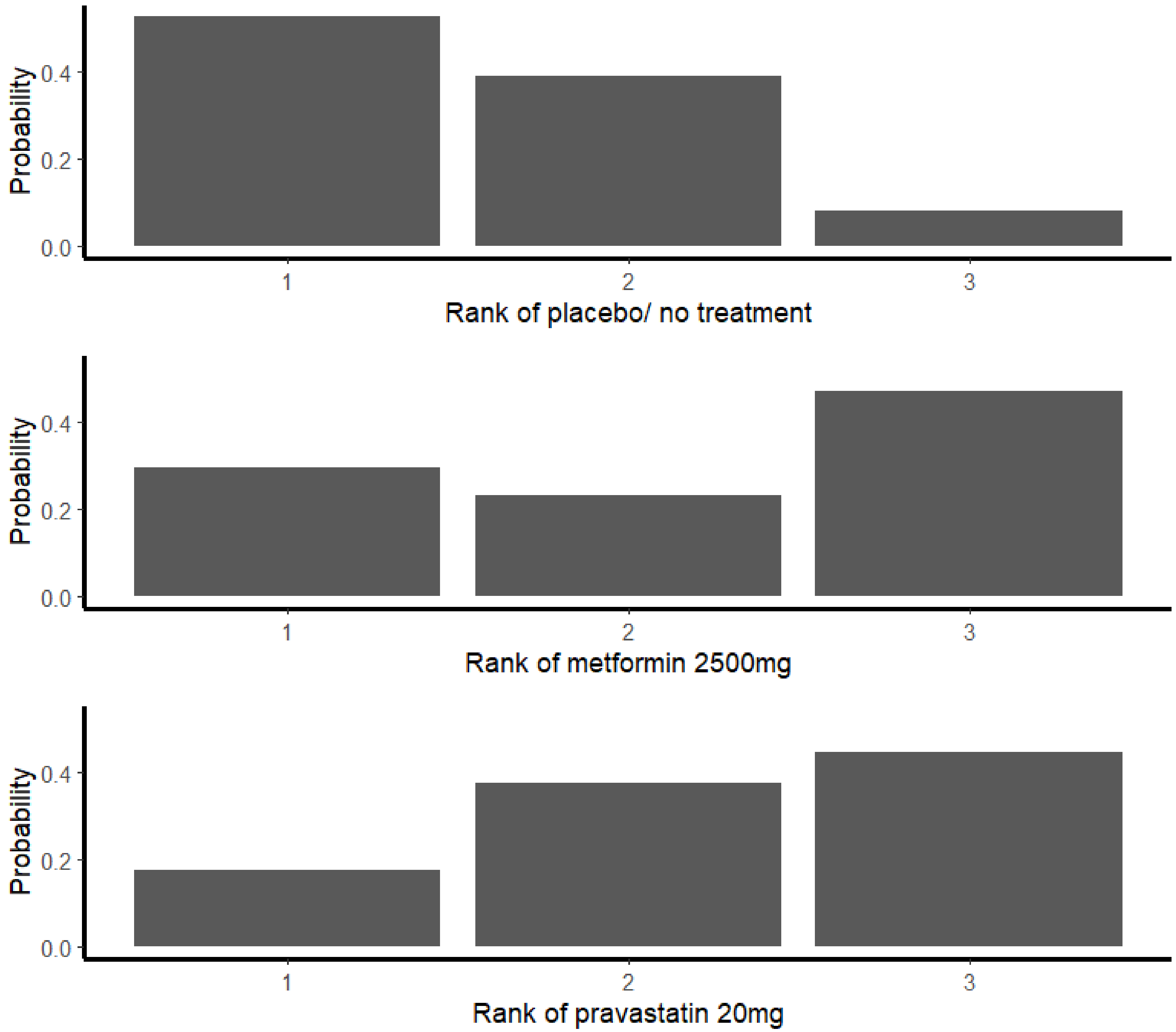

Figure 6 “Continue” shows Rankograms of neonates outcomes: (D) Respiratory distress syndrome RDS

E)

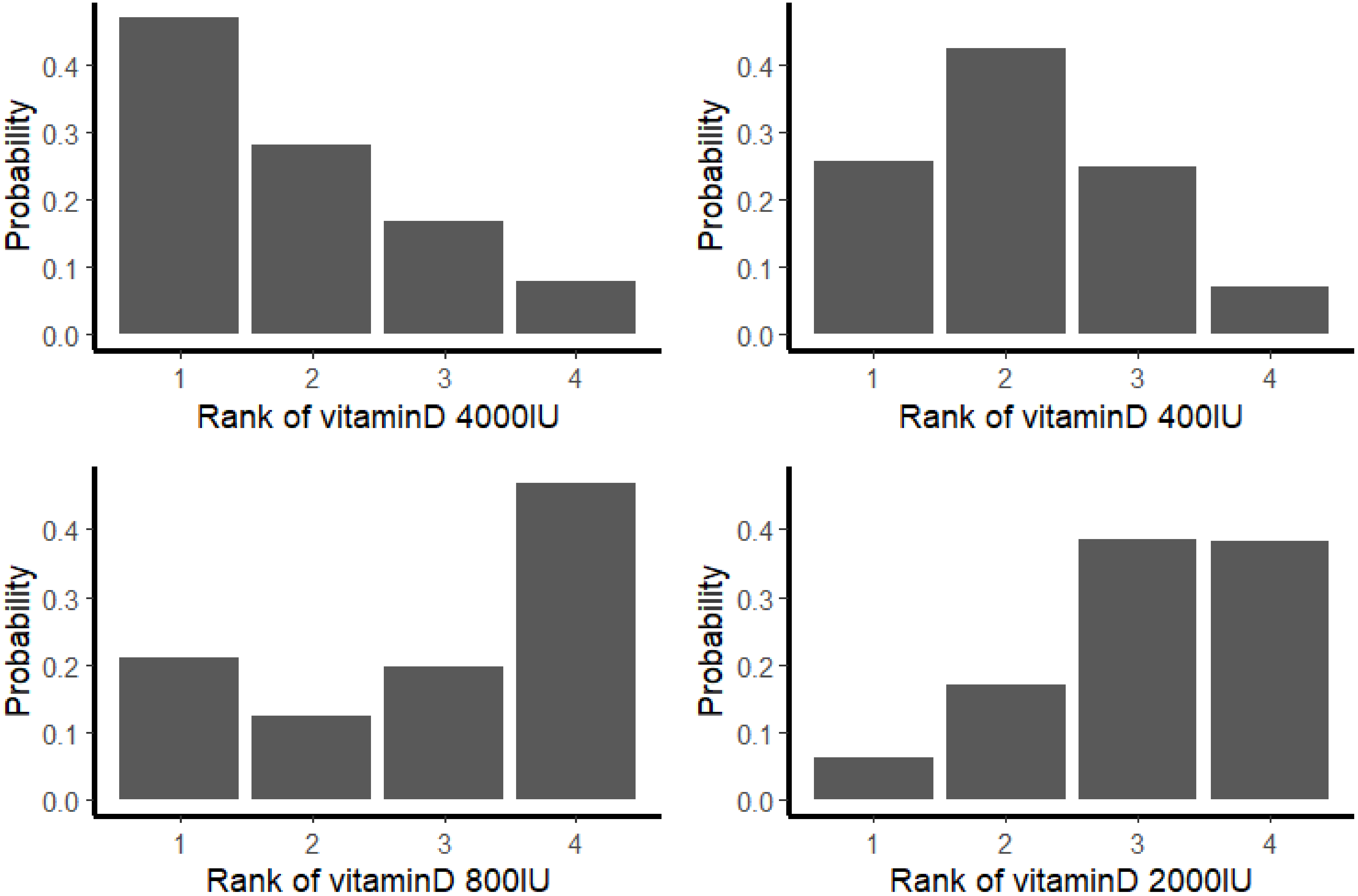

Figure 6 “Continue” shows Rankograms of neonates outcomes: (E) Low birth weight (vitamin D 400IU as comparator)

F)

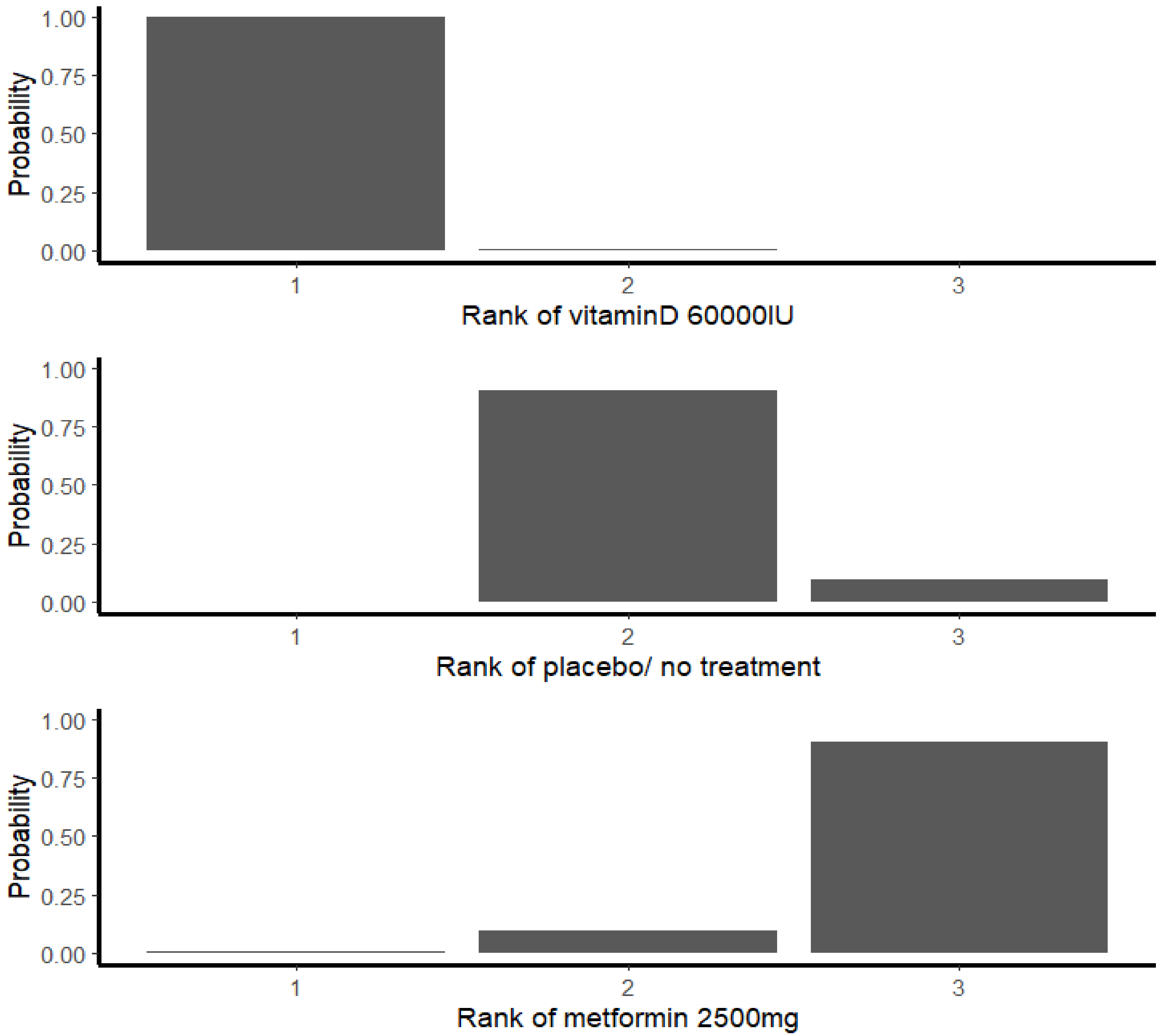

Figure 6 “Continue” shows Rankograms of neonates outcomes: (F) Low birth weight (placebo as comparator)

A)

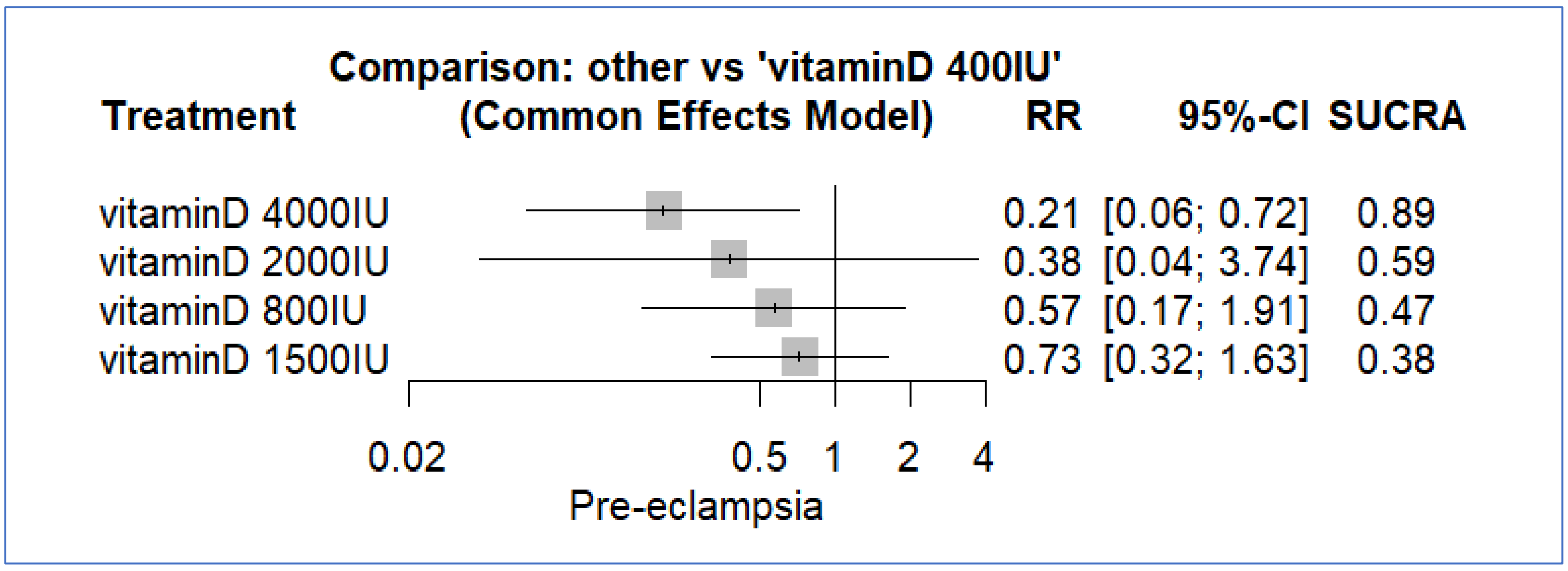

B)

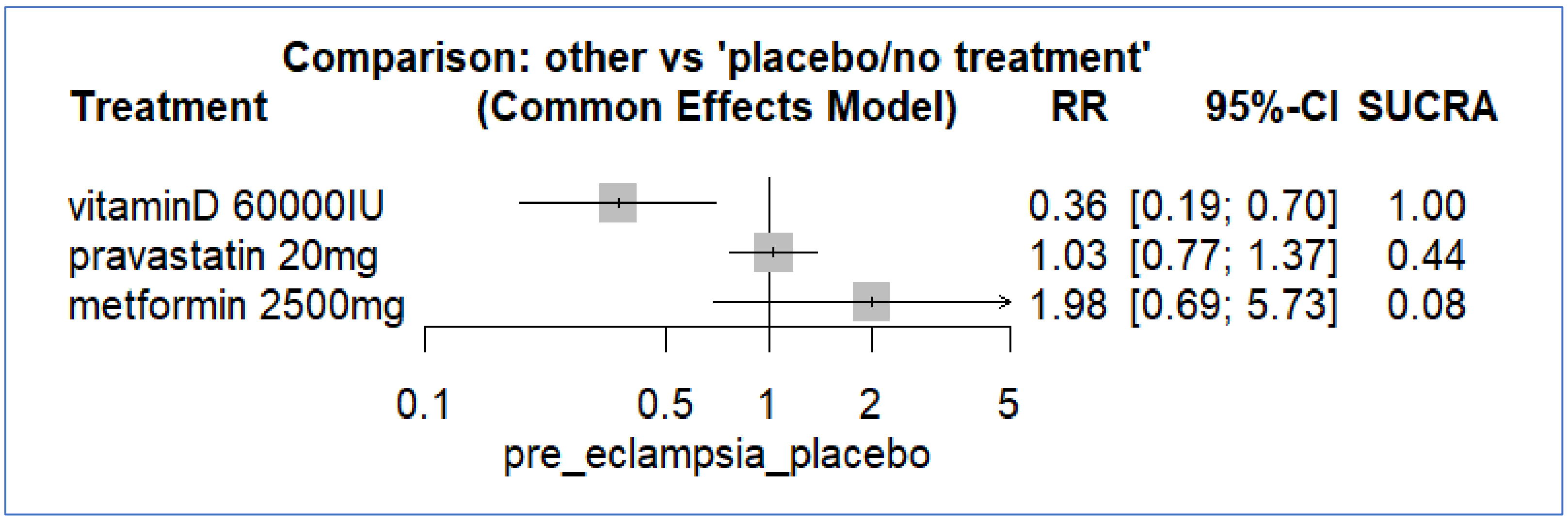

C)

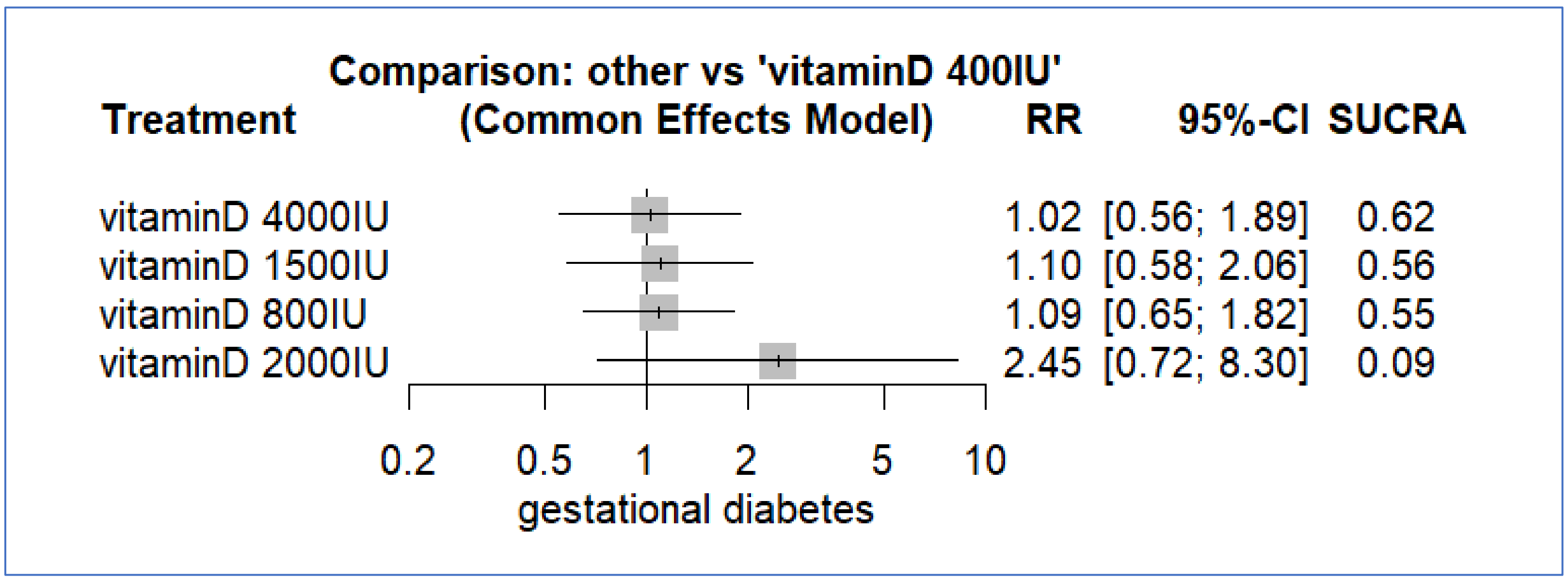

D)

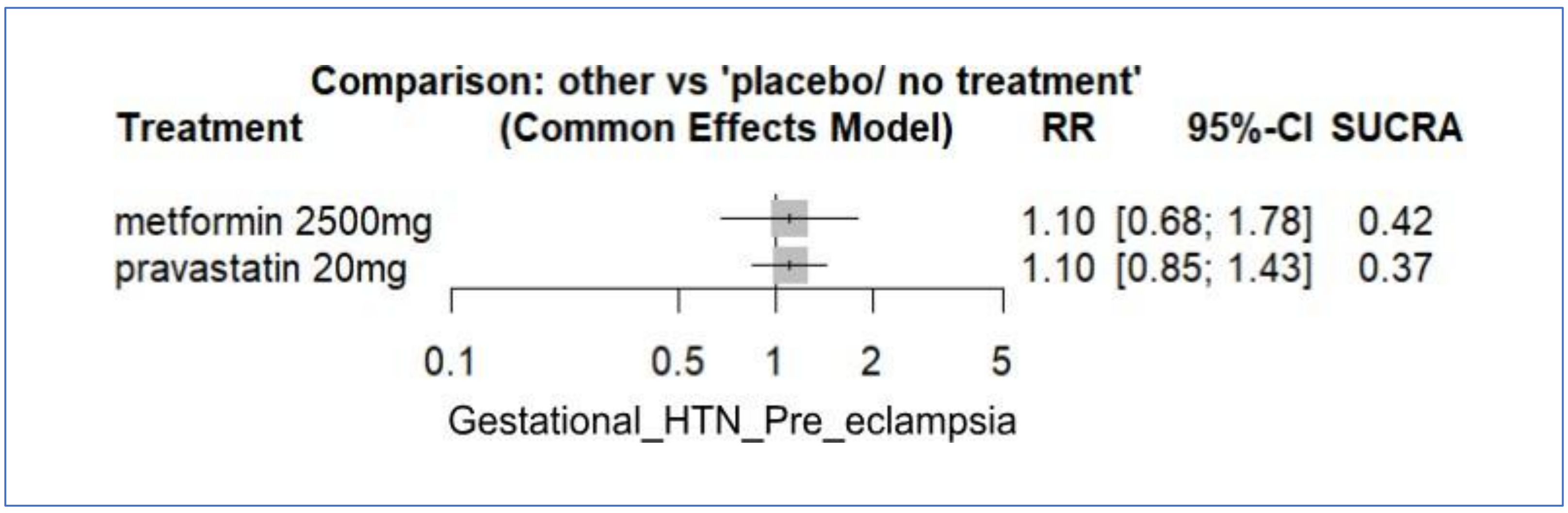

Figure 7 shows Forest plots presenting relative effect estimates from the network meta-analysis for maternal and neonatal outcomes. Effect sizes are expressed as risk ratios (RRs) with 95% confidence intervals, comparing each intervention with the reference comparator within each network: (A) Pre-eclampsia (vitamin D 400IU as comparator); (B) Pre-eclampsia (placebo as comparator); (C) Gestational diabetes; (D) Gestational hypertension

E)

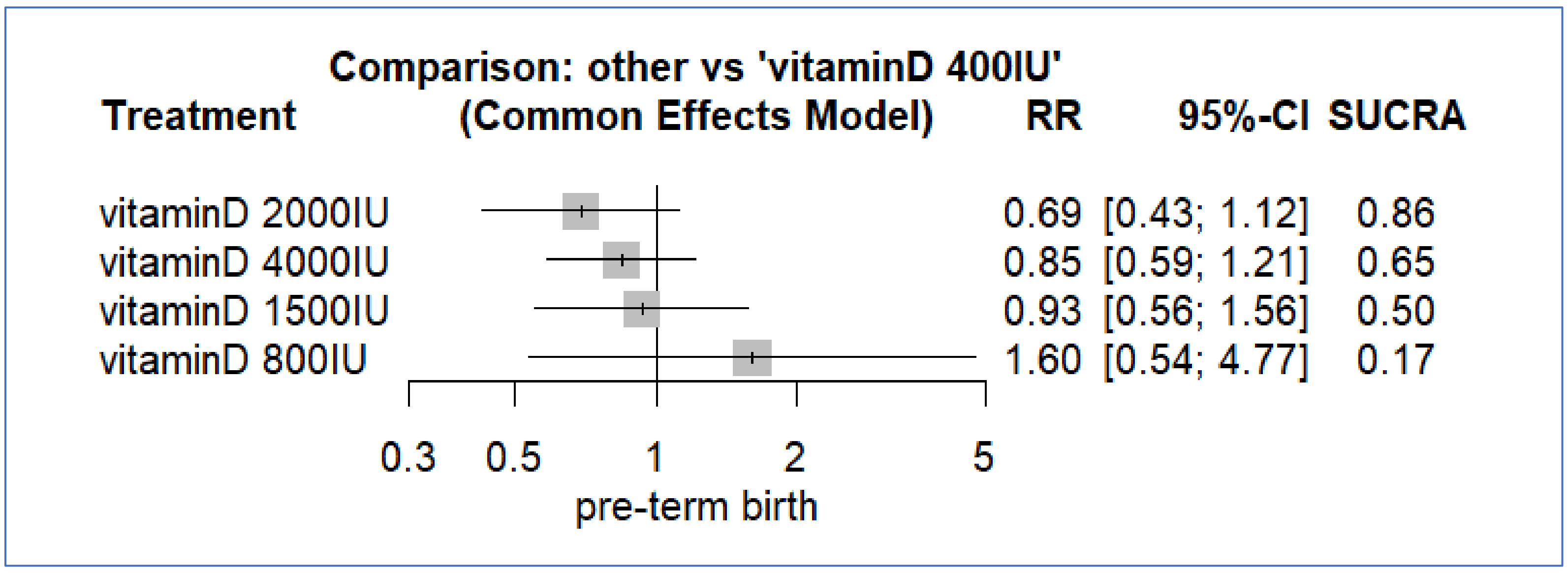

F)

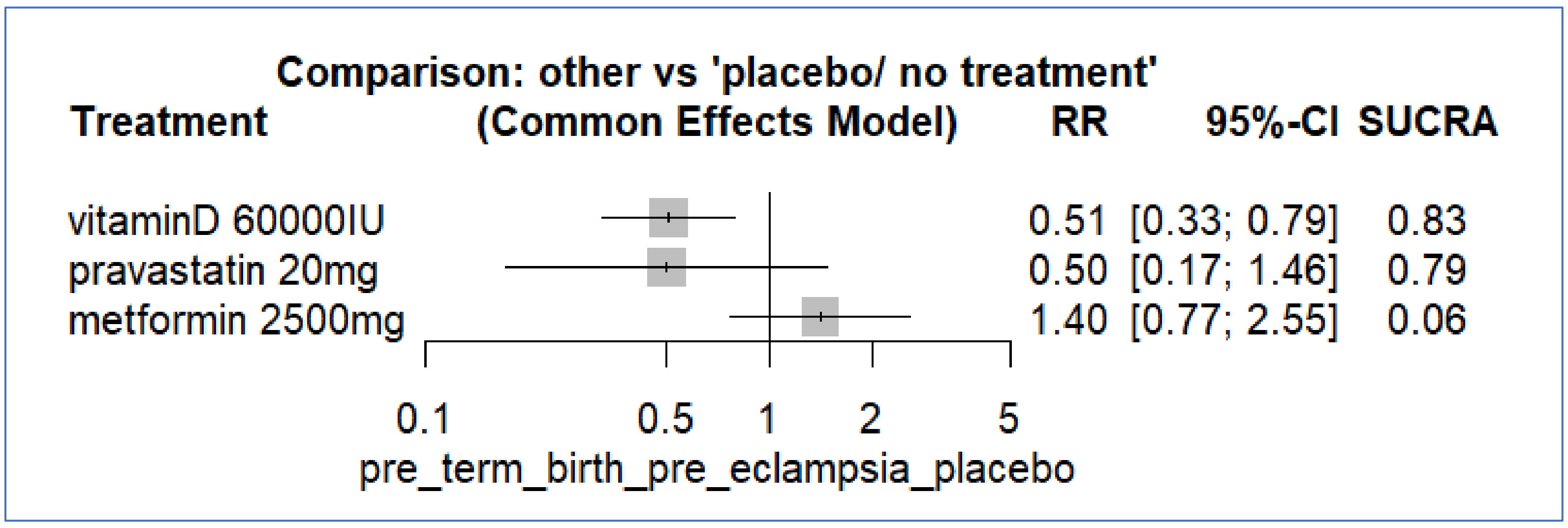

G)

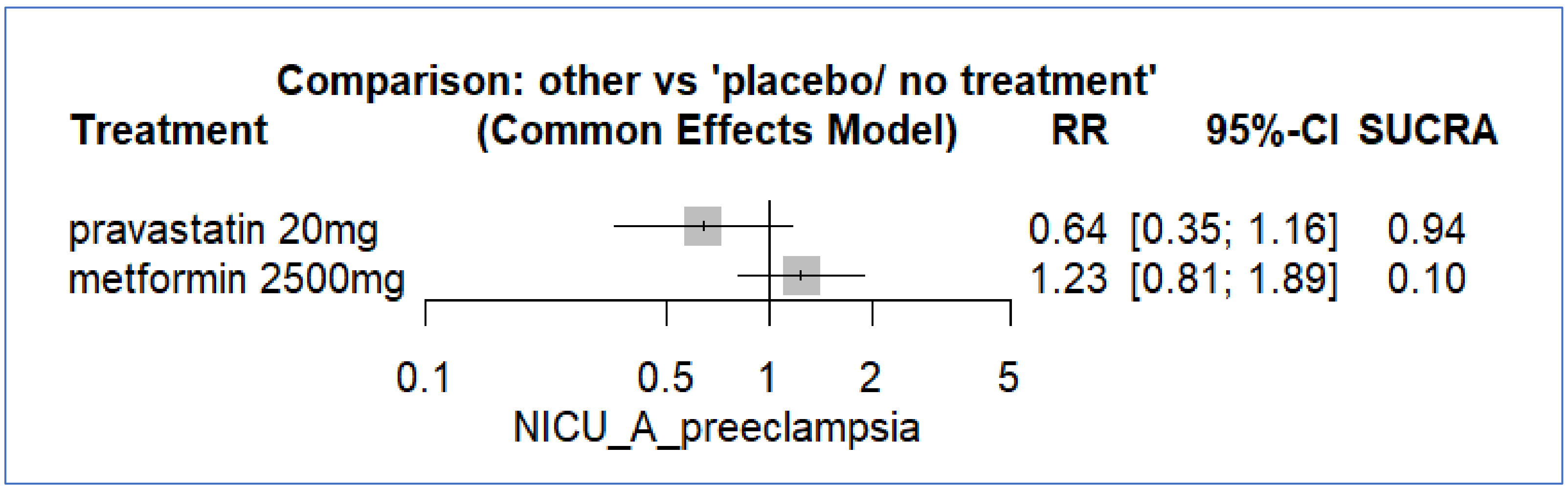

H)

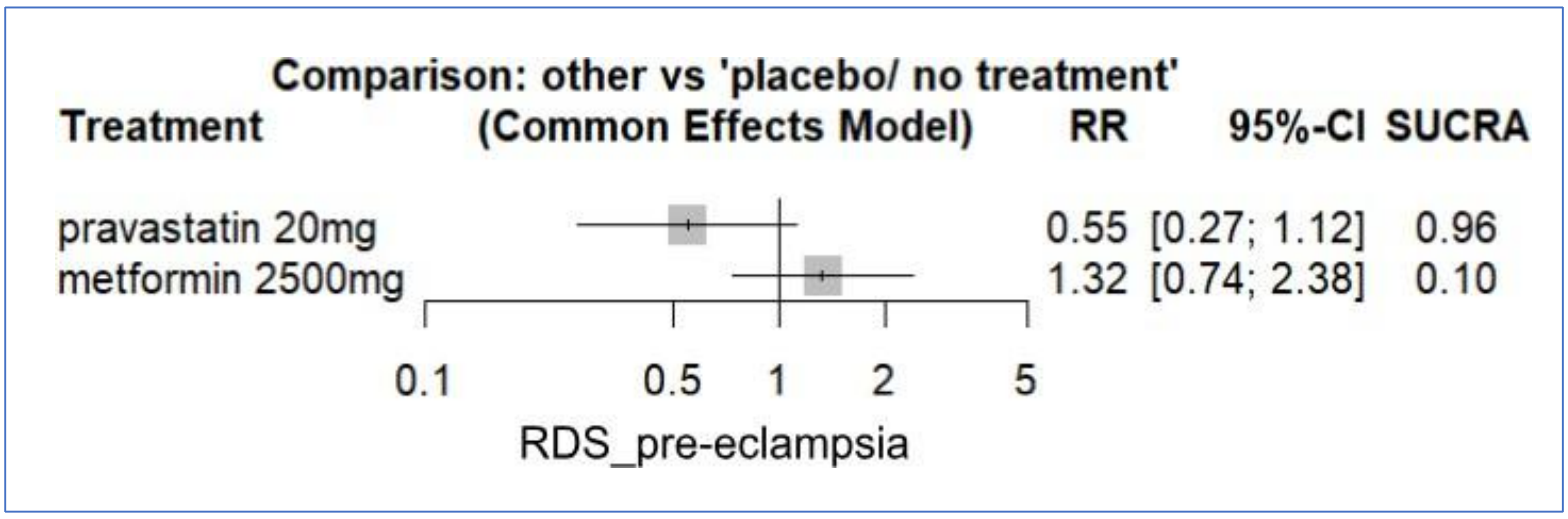

Figure 7 “Continue” shows Forest plots presenting relative effect estimates from the network meta-analysis for maternal and neonatal outcomes. Effect sizes are expressed as risk ratios (RRs) with 95% confidence intervals, comparing each intervention with the reference comparator within each network: (E) Pre-term birth (vitamin D 400IU as comparator); (F) Pre-term birth (placebo as comparator); (G) NICU admission; (H) Respiratory distress syndrome RDS

I)

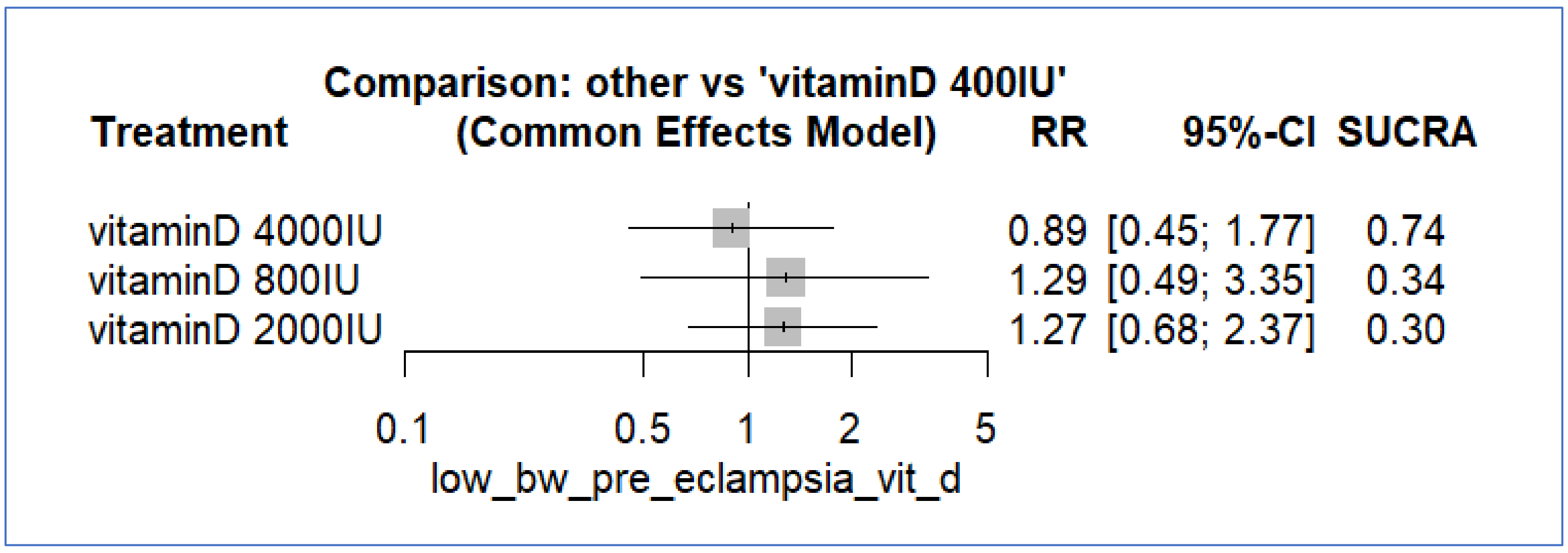

J)

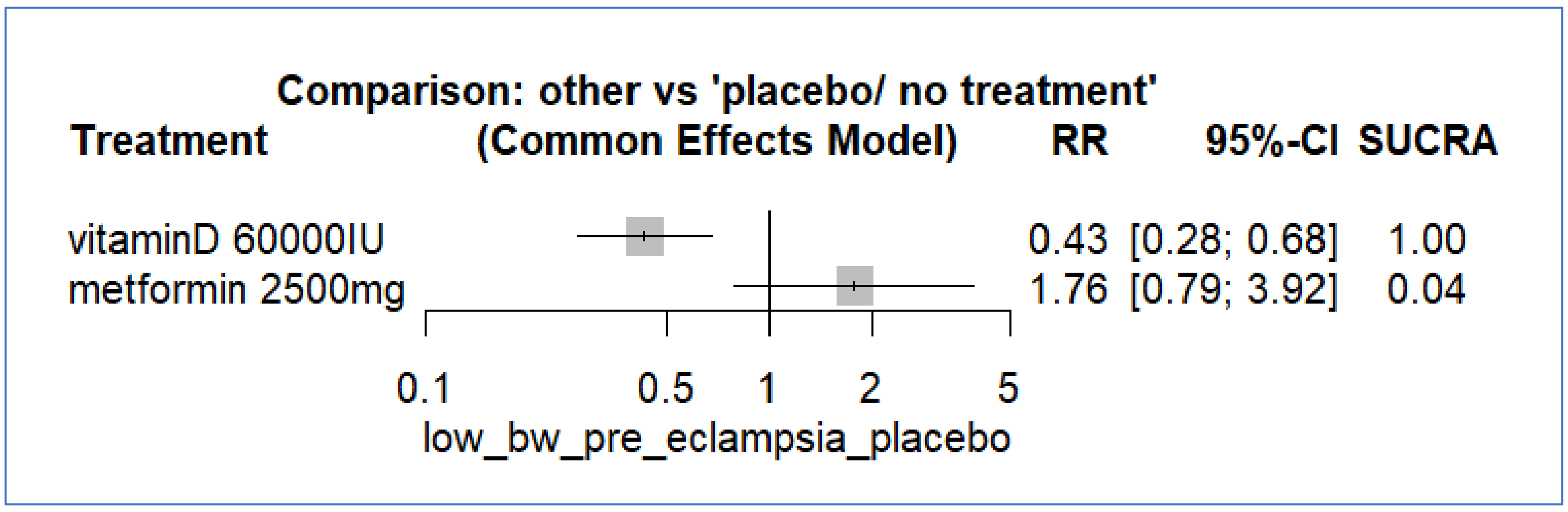

Figure 7 “Continue” shows Forest plots presenting relative effect estimates from the network meta-analysis for maternal and neonatal outcomes. Effect sizes are expressed as risk ratios (RRs) with 95% confidence intervals, comparing each intervention with the reference comparator within each network: (I) Low birth weight (vitamin D 400IU as comparator); (J) Low birth weight (placebo as comparator)
