# Supplementary material for: Comparative effectiveness of statins, metformin, and vitamin D, for prevention of preeclampsia: a systematic review and network meta-analysis of randomized trials
Source: BMC Pregnancy Childbirth. 2026 Oct 2;26:1101. doi: 10.1186/s12884-026-09920-6 (PMC13632318; doi:10.1186/s12884-026-09920-6)
Supplement: Supplementary file 3 [file 12884_2026_9920_MOESM4_ESM.docx]

| **Study ID** | **“High-risk” characteristics** | **Definitions of preeclampsia, gestational hypertension, preterm birth (threshold), and low birth weight/SGA** |
| --- | --- | --- |
| Kubayanaga, 2024 | Primigravid (nulliparous) women, a well‑established obstetric risk group for preeclampsia, enrolled in early pregnancy (≤16 weeks’ gestation) to target the placentation window relevant to preeclampsia pathogenesis. Vitamin D deficiency was not an inclusion criterion; however, baseline assessment showed a high prevalence of vitamin D insufficiency/deficiency, which was treated as a biological risk modifier rather than a defining eligibility condition. Women with chronic hypertension, diabetes mellitus, renal/hepatic disease, multiple gestation, or established preeclampsia at enrollment were excluded. | “Preeclampsia” was defined as hypertension (SBP≥140  and/or DBP≥90 mm Hg) associated with proteinuria measured by dipstick (≥2 crosses) at 20 GW.  "Preterm delivery" was defined as delivery occurring before 37 completed weeks of gestation.  Low birth weight was diagnosed in any newborn with a weight below the 10th  percentile for gestational age or less than 2500 g |
| Nausheen, 2021 | Pregnant women (<16 weeks’ gestation) from an urban Pakistani population with an extremely high baseline prevalence of vitamin D deficiency (97.4% deficient at enrolment), Women with major medical conditions (pre‑existing diabetes, chronic hypertension, chronic disease), multiple gestation, or fetal anomalies were excluded. * | “Preterm birth” is identified if birth occurs before 37 weeks gestation.  “Low birth weight” is defined as neonatal weight ≤2500 g at birth.  “Pre-eclampsia” is identified through blood pressure >140/90 mm Hg and proteinuria (≥300 mg). |
| Döbert, 2021 | High‑risk pregnancies were identified using a validated Bayesian survival‑time model that estimated each woman’s individualized risk of developing preeclampsia as a function of gestational age at delivery. Risk estimation combined maternal demographic and clinical factors (including age, weight, race, medical and obstetric history, method of conception, and family history) with biochemical and biophysical biomarkers measured at 35–37 weeks’ gestation. Biomarkers included mean arterial pressure, placental growth factor, and soluble fms‑like tyrosine kinase‑1, expressed as multiples of the median and adjusted for relevant maternal and technical factors. Women were classified as high risk based on the posterior probability derived from this integrated model rather than a single clinical criterion. | Preeclampsia, defined according to the American College of Obstetricians and Gynecologists (ACOG) criteria, includes new‑onset hypertension after 20 weeks’ gestation (systolic blood pressure ≥140 mmHg or diastolic blood pressure ≥90 mmHg on two occasions) with proteinuria, or in the absence of proteinuria, hypertension accompanied by maternal end‑organ dysfunction. Diagnosis of preeclampsia underwent centralized adjudication. Secondary outcomes included adverse pregnancy outcomes at any gestational age and at ≥37 weeks, stillbirth or neonatal death, neonatal morbidity and need for neonatal therapy, and low birth weight. |
| Dunne, 2023 | gestational diabetes according to the World Health Organization 2013 (based on any one of the following glucose values: fasting ≥5.1 mmol/L, 1 hour ≥10 mmol/L, or 2 hours ≥8.5 mmol/L). | Gestational hypertension and preeclampsia were reported, but no explicit diagnostic definitions were provided.  Secondary neonatal outcomes included infant birthweight, length, and head circumference with derived measures for large for gestational age above the 90th percentile, small for gestational age below the 10th percentile, macrosomia greater than 4 kg, low birth weight less than 2.5 kg, proportion of infants with neonatal morbidities, including need for neonatal intensive care, respiratory distress requiring respiratory support,  jaundice requiring phototherapy, major congenital anomalies, Apgar score less than 7 at 5 minutes, and neonatal hypo-  glycemia (<2.6 mmol/L on 1 or more occasions within 60 minutes postdelivery). |
| Constantine, 2021 | a history of preeclampsia with severe features in a pregnancy that required delivery prior to 34 6/7 weeks of gestation | Preeclampsia was diagnosed according to criteria set by the American  College of Obstetricians and Gynecologists (ACOG), including new‑onset hypertension after 20 weeks’ gestation (systolic blood pressure ≥140 mmHg or diastolic blood pressure ≥90 mmHg on two occasions) with proteinuria, or in the absence of proteinuria, hypertension accompanied by maternal end‑organ dysfunction, the diagnosis (or absence) was confirmed  by a panel of maternal-fetal medicine physicians. |
| Manasova, 2021 | presence of Vitamin D insufficiency/deficiency, as well as the presence of the following risk factors for the development of preeclampsia (according to the  recommendations of ACOG 2020—the  forthcoming first birth, multiple pregnancy, preeclampsia (PE) in a previous pregnancy,  chronic hypertension, pre-existing or gestational  diabetes, thrombophilia, systemic lupus  erythematosus, body mass index over 30 kg/m²  ,antiphospholipid syndrome, maternal age 35 years  old or more, kidney disease, use of assisted  reproductive technologies, or indication of  obstructive sleep apnea. To determine the vitamin D status, we used the recommendations of Polish colleagues (2019), according to which VD deficiency was stated at a blood level of 25 (OH) D less than 30 ng/ml [9]. The level of the circulating metabolite of vitamin D, total for chole- and ergocalciferol, (25 (OH) D), was determined by the enzyme-linked  immunosorbent assay. | The criterion for the diagnosis of PE was  considered the level of systolic blood pressure (SBP) 140 mm Hg or more or diastolic blood pressure (DBP) 90 mm Hg or more with 2-fold measurement within 4 hours after 20 weeks of pregnancy in women with previous normal blood pressure; with a systolic blood pressure of 160 mm Hg or more, or a systolic blood pressure of 110 mm Hg or more, a single blood pressure measurement was sufficient for the timely initiation of antihypertensive therapy.  The diagnosis of PE was confirmed by proteinuria in the middle portion of urine collected twice with an interval of 4 hours or more at a protein content of  0.3 g/L or a daily protein excretion of 0.3 g, dysfunction, and other criteria for PE. |
| Xiaomang, 2020 | All pregnant women had a clinically  definitive diagnosis of vitamin D deficiency as the 25 [OH] D indicator is lower than 25 nmol/L, | The criteria for pre-eclampsia are headache, vertigo, nausea, vomiting,  epigastric discomfort symptoms, edema, proteinuria, and systolic blood pressure >160 mmHg. At the same time. |
| Ku, 2023 | Overweight and obese women with a pre-pregnancy  BMI of ≥25 kg/m² and were within 16 weeks of gestation. Individuals with specific current or past medical histories, as identified at recruitment, were excluded. These included hypo/hyperparathyroidism, hypercalciuria, hypercalcemia, osteomalacia, liver dysfunction, tuberculosis, renal disease, and sarcoidosis. Individuals with multiple pregnancies, pre-existing diabetes mellitus, diagnosed gestational diabetes mellitus (GDM) or gestational hypertension at recruitment, chronic hypertension, or use of lipid-lowering medication were also excluded. | Preeclampsia was defined as new‑onset hypertension after 20 weeks’ gestation (systolic blood pressure ≥140 mmHg and/or diastolic blood pressure ≥90 mmHg) accompanied by proteinuria (≥0.3 g protein in a 24‑hour urine collection), consistent with international guideline‑based definitions used at the time of the trial. Gestational hypertension was defined as systolic blood pressure ≥140 mmHg and/or diastolic blood pressure ≥90 mmHg without accompanying proteinuria. Preterm birth was defined as delivery before 37 completed weeks of gestation. Low birth weight was defined as birth weight <2500 g. |

*This trial represents a biologically high‑risk pregnancy population due to near‑universal baseline vitamin D deficiency, despite the absence of selection based on classical obstetric comorbidities.

*Outcome definitions are based on information available in the published trial reports. In studies where detailed definitions were not provided, outcomes relied on the trial authors’ reporting, without additional assumptions by the review authors.
