# Supplementary material for: Comparative effectiveness of statins, metformin, and vitamin D, for prevention of preeclampsia: a systematic review and network meta-analysis of randomized trials
Source: BMC Pregnancy Childbirth. 2026 Oct 2;26:1101. doi: 10.1186/s12884-026-09920-6 (PMC13632318; doi:10.1186/s12884-026-09920-6)
Supplement: Supplementary file 4 [file 12884_2026_9920_MOESM5_ESM.docx]

**List for research strategy for databases included in the study**

1. **Pubmed**

**Search strategy used :**

(("Pre-Eclampsia"[Mesh] OR "Hypertension, Pregnancy-Induced"[Mesh]

OR preeclampsia[tiab] OR pre-eclampsia[tiab] OR preeclamptic[tiab]

OR "gestational hypertension"[tiab]))

AND

(("Pregnancy"[Mesh]

OR pregnan*[tiab] OR antenatal[tiab] OR "high risk pregnancy"[tiab] OR "high-risk pregnan*"[tiab]))

AND

(("Hydroxymethylglutaryl-CoA Reductase Inhibitors"[Mesh]

OR statin*[tiab] OR pravastatin[tiab] OR simvastatin[tiab] OR atorvastatin[tiab]

OR rosuvastatin[tiab] OR fluvastatin[tiab] OR lovastatin[tiab])

OR

("Metformin"[Mesh] OR metformin[tiab])

OR

("Vitamin D"[Mesh] OR cholecalciferol[tiab] OR calcifediol[tiab] OR ergocalciferol[tiab] OR "vitamin d3"[tiab])

OR

("Fatty Acids, Omega-3"[Mesh] OR "Fish Oils"[Mesh]

OR "omega 3"[tiab] OR "omega-3"[tiab] OR "fish oil"[tiab] OR EPA[tiab] OR DHA[tiab] OR "n-3 fatty acid*"[tiab]))

AND

(("Infant, Small for Gestational Age"[Mesh] OR "Infant, Low Birth Weight"[Mesh]

OR "Premature Birth"[Mesh] OR "Infant, Premature"[Mesh] OR "Perinatal Mortality"[Mesh]

OR "Intensive Care Units, Neonatal"[Mesh]

OR "Congenital Abnormalities"[Mesh]

OR "Maternal Mortality"[Mesh] OR "Pregnancy Complications"[Mesh]

OR "Treatment Outcome"[Mesh]

OR "preterm birth"[tiab] OR "early-onset preeclampsia"[tiab] OR "gestational hypertension"[tiab]

OR "small for gestational age"[tiab] OR SGA[tiab] OR IUGR[tiab]

OR "perinatal mortality"[tiab] OR "NICU admission"[tiab]

OR "maternal adverse event*"[tiab] OR "congenital anomaly*"[tiab] OR "neonatal adverse event*"[tiab]))

AND

(("Randomized Controlled Trial"[Publication Type]

OR "Randomized Controlled Trials as Topic"[Mesh]

OR "Clinical Trials as Topic"[Mesh]

OR random*[tiab] OR "clinical trial"[tiab])

**Date of access :** 9/11/2025

**Filters applied :** RCTs + last 5 years (from 9/11/2020 to 9/11/2025)

**Total studies : 21**

2. **Epistemonikos**

**Search strategy used :**

((preeclampsia OR "pre-eclampsia" OR preeclampsi* OR preeclamptic OR "gestational hypertension")

AND (pregnan* OR "pregnant women" OR antenatal OR "high risk pregnancy" OR "high-risk pregnan*"))

AND

((statin* OR pravastatin OR simvastatin OR atorvastatin OR rosuvastatin OR fluvastatin OR lovastatin OR "HMG-CoA reductase inhibitor*")

OR (metformin)

OR ("vitamin D" OR cholecalciferol OR calcifediol OR ergocalciferol OR "vitamin d3")

OR ("omega 3" OR "omega-3" OR "fish oil" OR EPA OR DHA OR "n-3 fatty acid*"))

AND

("preterm birth" OR "early-onset preeclampsia" OR "gestational hypertension" OR "small for gestational age" OR SGA OR IUGR

OR "perinatal mortality" OR "NICU admission" OR "maternal adverse event*" OR "congenital anomaly*" OR "neonatal adverse event*")

AND

(random* OR "randomized controlled trial" OR "randomised controlled trial" OR "clinical trial")

**Date of access :** 9/10/2025

**Filters applied :** Last 5 years (from 9/11/2020 to 9/11/2025)

**Total studies :** 71

3. **Scopus**

**Search strategy used :**

((preeclampsia OR "pre-eclampsia" OR preeclampsi* OR preeclamptic OR "gestational hypertension")

AND (pregnan* OR "pregnant women" OR antenatal OR "high risk pregnancy" OR "high-risk pregnan*"))

AND

((statin* OR pravastatin OR simvastatin OR atorvastatin OR rosuvastatin OR fluvastatin OR lovastatin OR "HMG-CoA reductase inhibitor*")

OR (metformin)

OR ("vitamin D" OR cholecalciferol OR calcifediol OR ergocalciferol OR "vitamin d3")

OR ("omega 3" OR "omega-3" OR "fish oil" OR EPA OR DHA OR "n-3 fatty acid*"))

AND

("preterm birth" OR "early-onset preeclampsia" OR "gestational hypertension" OR "small for gestational age" OR SGA OR IUGR

OR "perinatal mortality" OR "NICU admission" OR "maternal adverse event*" OR "congenital anomaly*" OR "neonatal adverse event*")

AND

(random* OR "randomized controlled trial" OR "randomised controlled trial" OR "clinical trial")

**Date of access :** 9/11/2025

**Filters applied :** Last 5 years (from 9/11/2020 to 9/11/2025)

**Total studies :** 125

4. **Web of Science**

**Search strategy used :**

((preeclampsia OR "pre-eclampsia" OR preeclampsi* OR preeclamptic OR "gestational hypertension")

AND (pregnan* OR "pregnant women" OR antenatal OR "high risk pregnancy" OR "high-risk pregnan*"))

AND

((statin* OR pravastatin OR simvastatin OR atorvastatin OR rosuvastatin OR fluvastatin OR lovastatin OR "HMG-CoA reductase inhibitor*")

OR (metformin)

OR ("vitamin D" OR cholecalciferol OR calcifediol OR ergocalciferol OR "vitamin d3")

OR ("omega 3" OR "omega-3" OR "fish oil" OR EPA OR DHA OR "n-3 fatty acid*"))

AND

("preterm birth" OR "early-onset preeclampsia" OR "gestational hypertension" OR "small for gestational age" OR SGA OR IUGR

OR "perinatal mortality" OR "NICU admission" OR "maternal adverse event*" OR "congenital anomaly*" OR "neonatal adverse event*")

AND

(random* OR "randomized controlled trial" OR "randomised controlled trial" OR "clinical trial")

**Date of access :** 9/11/2025

**Filters applied :** Articles + Last 5 years (from 9/11/2020 to 9/11/2025)

**Total studies :** 35

5. **Cochrane**

**Search strategy used :**

(preeclampsia OR pre-eclampsia OR gestational hypertension) AND (pregnan* OR antenatal) AND (statin* OR pravastatin OR metformin OR "vitamin D" OR cholecalciferol OR "omega-3" OR "fish oil") AND ("preterm birth" OR "early-onset preeclampsia" OR SGA OR IUGR OR "perinatal mortality" OR "NICU admission") AND (random* OR "randomized controlled trial" OR "clinical trial")

**Date of access :** 9/11/2025

**Filters applied :** Trials + Last 5 years (September 11, 2020, to September 11, 2025 )

**Total studies :** 10
